# A Randomized Trial of Deferred Stenting Versus Immediate Stenting to Prevent No- or Slow-Reflow in Acute ST-Segment Elevation Myocardial Infarction (DEFER-STEMI)

**DOI:** 10.1016/j.jacc.2014.02.530

**Published:** 2014-05-27

**Authors:** David Carrick, Keith G. Oldroyd, Margaret McEntegart, Caroline Haig, Mark C. Petrie, Hany Eteiba, Stuart Hood, Colum Owens, Stuart Watkins, Jamie Layland, Mitchell Lindsay, Eileen Peat, Alan Rae, Miles Behan, Arvind Sood, W. Stewart Hillis, Ify Mordi, Ahmed Mahrous, Nadeem Ahmed, Rebekah Wilson, Laura Lasalle, Philippe Généreux, Ian Ford, Colin Berry

**Affiliations:** ∗ BHF Glasgow Cardiovascular Research Center, Institute of Cardiovascular and Medical Sciences, University of Glasgow, Glasgow, United Kingdom; † West of Scotland Heart and Lung Center, Golden Jubilee National Hospital, Dunbartonshire, United Kingdom; ‡ Robertson Center for Biostatistics, University of Glasgow, Glasgow, United Kingdom; § Royal Infirmary of Edinburgh, Edinburgh, United Kingdom; ‖ Hairmyres Hospital, Lanarkshire, United Kingdom; ¶ Cardiovascular Research Foundation and Columbia University Medical Center, New York, New York

**Keywords:** deferred stenting, myocardial infarction, myocardial salvage, no-reflow, primary percutaneous coronary intervention, ECG, electrocardiogram, IQR, interquartile range, MRI, magnetic resonance imaging, PCI, percutaneous coronary intervention, STEMI, ST-segment elevation myocardial infarction, TIMI, Thrombolysis In Myocardial Infarction

## Abstract

**Objectives:**

The aim of this study was to assess whether deferred stenting might reduce no-reflow and salvage myocardium in primary percutaneous coronary intervention (PCI) for ST-segment elevation myocardial infarction (STEMI).

**Background:**

No-reflow is associated with adverse outcomes in STEMI.

**Methods:**

This was a prospective, single-center, randomized, controlled, proof-of-concept trial in reperfused STEMI patients with ≥1 risk factors for no-reflow. Randomization was to deferred stenting with an intention-to-stent 4 to 16 h later or conventional treatment with immediate stenting. The primary outcome was the incidence of no-/slow-reflow (Thrombolysis In Myocardial Infarction ≤2). Cardiac magnetic resonance imaging was performed 2 days and 6 months after myocardial infarction. Myocardial salvage was the final infarct size indexed to the initial area at risk.

**Results:**

Of 411 STEMI patients (March 11, 2012 to November 21, 2012), 101 patients (mean age, 60 years; 69% male) were randomized (52 to the deferred stenting group, 49 to the immediate stenting). The median (interquartile range [IQR]) time to the second procedure in the deferred stenting group was 9 h (IQR: 6 to 12 h). Fewer patients in the deferred stenting group had no-/slow-reflow (14 [29%] vs. 3 [6%]; p = 0.006), no reflow (7 [14%] vs. 1 [2%]; p = 0.052) and intraprocedural thrombotic events (16 [33%] vs. 5 [10%]; p = 0.010). Thrombolysis In Myocardial Infarction coronary flow grades at the end of PCI were higher in the deferred stenting group (p = 0.018). Recurrent STEMI occurred in 2 patients in the deferred stenting group before the second procedure. Myocardial salvage index at 6 months was greater in the deferred stenting group (68 [IQR: 54% to 82%] vs. 56 [IQR: 31% to 72%]; p = 0.031].

**Conclusions:**

In high-risk STEMI patients, deferred stenting in primary PCI reduced no-reflow and increased myocardial salvage. (Deferred Stent Trial in STEMI; NCT01717573)

Primary percutaneous coronary intervention (PCI) with stenting immediately after coronary reperfusion salvage procedures jeopardizes myocardium, improves prognosis, and is the current standard of care for acute ST-segment elevation myocardial infarction (STEMI) 1, 2. No-reflow is defined as an acute reduction in myocardial blood flow despite a patent epicardial coronary artery (3). The pathophysiology of no-reflow involves microvascular obstruction secondary to distal embolization of clot, microvascular spasm, and thrombosis (3). No-reflow occurs in ∼10% of cases of primary PCI and is associated with patient characteristics such as advanced age and delayed presentation and coronary characteristics such as a completely occluded culprit artery and heavy thrombus burden 3, 4, 5, 6, 7.

No therapies have been shown to prevent no-reflow, and when it occurs, treatment by administration of vasodilator drugs (8) and intra-aortic balloon counterpulsation therapy is empirical 2, 3, 8, 9. The rationale for our intervention was to avoid the potential adverse effects of immediate stenting when the likelihood of no-reflow might be greatest. Deferred stent implantation might allow time for reduction in coronary thrombus burden and recovery of microvascular function such that the likelihood of no-reflow is reduced. We hypothesized that after initial coronary reperfusion and normalization of coronary blood flow, brief deferral of stenting might reduce the occurrence of no-reflow compared with usual care with immediate stenting and increase myocardial salvage. We investigated this hypothesis in a real-life clinical setting involving STEMI patients treated with primary PCI.

## Methods

### Trial design

We performed a prospective, randomized, controlled, parallel group trial in STEMI patients enrolled at a single center between March 11, 2012, and November 21, 2012. The trial was a proof-of-concept trial nested in a larger prospective cohort study (10).

### Participants and eligibility criteria

Patients at risk of no-reflow were selected if radial artery access was used and ≥1 of the following inclusion criteria were met: 1) clinical history 3, 4, 5, 6, 7 that included myocardial infarction, increased age (i.e., 65 years of age or older), duration of symptoms >6 h; 2) culprit coronary artery abnormalities 3, 4, 5, 6, 7 including an occluded artery (Thrombolysis In Myocardial Infarction [TIMI] flow grade 0/1 [11]) at initial angiography, heavy thrombus burden (TIMI grade 2 or higher [12]), long lesion length (≥24 mm), small vessel diameter (i.e., ≤2.5 mm); 3) clinical signs of acute microvascular injury after initial reperfusion 3, 4, 5, 6, 7 with persistent ST-segment elevation >50%.

The exclusion criteria were 1) the absence of normal (TIMI flow grade 3) coronary blood flow after initial reperfusion with aspiration thrombectomy with or without balloon angioplasty. The residual severity of the culprit stenosis was not relevant to participation provided TIMI flow grade 3 was evident; 2) cardiogenic shock; 3) a contraindication to magnetic resonance imaging (MRI) (e.g., permanent pacemaker); 4) inability to give informed consent.

### Setting

Consecutive STEMI patient admissions were screened for these inclusion and exclusion criteria. During ambulance transfer to the hospital, the patients received 300 mg aspirin, 600 mg clopidogrel, and 5000 IU unfractionated heparin 2, 9. Catheter laboratory management is described in the Online Appendix.

### Informed consent

Witnessed informed consent was verbally obtained after coronary reperfusion in eligible patients in the cardiac catheter laboratory. When the patient returned to the Coronary Care Unit, a patient information sheet approved by the local ethics committee was provided, and written informed consent was then obtained. The patients who were not randomized were included in a registry.

### Randomization, implementation, and blinding

Randomization took place immediately after obtaining verbal consent using a Web-based computer tool with a concealed random allocation sequence provided by the independent clinical trials unit and implemented by the catheter laboratory physiologist. Randomization was on a 1:1 basis between usual care with immediate stenting and deferred stenting.

### Interventions

The deferred PCI strategy involved an intention-to-stent 4 to 16 h after initial coronary reperfusion. This time interval was based on a balance between competing benefits and risks. A short minimum period (4 h) was adopted, given our concern about the theoretical time-related risk of coronary reocclusion. In practice, a guideline of at least 8 h was recommended for the deferred PCI to permit the beneficial effects of reperfusion and antithrombotic therapies and so that all patients could be treated between 7:00 am and 11 pm during the first 24 h of admission to ensure that the second procedure occurred at a time that facilitated a rest period for the patient and the staff. Finally, an upper limit of 16 h was set to minimize any prolongation of the hospital admission.

The treatment protocol for deferred patients included transfer to the Coronary Care Unit, continuous intravenous infusion of glycoprotein IIb/IIIa inhibitor therapy (tirofiban 0.15 μg/kg/min) and administration of subcutaneous low molecular weight heparin (enoxaparin 1 mg/kg every 12 h) for up to 16 h. The radial artery sheath used for PCI was retained or removed according to operator and patient preference. Arterial blood pressure and the radial sheath site were monitored in the Coronary Care Unit. All patients also had continuous electrocardiographic monitoring in the Coronary Care Unit.

Usual care included immediate stenting in the catheter laboratory and intravenous glycoprotein IIb/IIIa inhibitor therapy for 12 h (tirofiban 0.15 μg/kg/min). After the PCI procedure was completed, the patients returned to the Coronary Care Unit and were treated with optimal secondary prevention measures (2).

### Outcomes

#### Primary Outcome

The primary outcome was the incidence of no-/slow-reflow (2), defined as absent flow (TIMI flow grade 0), incomplete filling (TIMI flow grade 1), or slow-reflow but complete filling (TIMI flow grade 2) of the culprit coronary artery during or at the end of the PCI as revealed by the coronary angiogram. Both angiograms were analyzed for the deferred stenting group and the worst/lowest flow grade and myocardial blush grade were recorded. The definition of no-reflow also required the absence of coronary dissection or obstruction (e.g., due to thrombus) that could cause a decrease in coronary blood flow (3).

#### Secondary Outcomes

The secondary outcomes included angiographic, electrocardiographic, and MRI parameters.

#### Angiographic Secondary Outcomes

The angiographic secondary outcomes were no-reflow (TIMI flow grade 0/1), final TIMI flow grade (11), corrected TIMI frame count (12) TIMI myocardial blush grade (13), the occurrence of intraprocedural thrombotic events (14) (defined as the development of new or increasing thrombus, abrupt vessel closure, or distal embolization occurring at any time during the procedure in the culprit vessel or any significant side branch measuring ≥2 mm). Embolization was defined as a distal filling defect with an abrupt cutoff in one of the peripheral coronary artery branches of the infarct-related vessel, distal to the site of angioplasty (9). In the deferred stenting group, the intended stent strategy at the end of the first procedure was prospectively recorded, and stent dimensions were compared with the actual stents used in the second procedure by the same operator (Online Appendix). In addition, thrombus burden at the start of the second procedure was compared with the end of the first procedure. Thrombus area was delineated as a contrast filling defect using QCA software (Medis QAngio XA software v. 7.2.34., Raleigh, North Carolina) and expressed in square millimeters.

#### Electrocardiographic Secondary Outcomes

The electrocardiographic secondary outcomes included the occurrence of complete (≥70), partial (30% to <70%), or no (≤30%) ST-segment resolution on the electrocardiogram (ECG) assessed 60 min after reperfusion compared with the baseline ECG before reperfusion 2, 9. In addition, ST-segment elevation was measured on the baseline ECG before reperfusion to estimate the extent of initial myocardial jeopardy with the Aldrich ST-segment elevation score (15).

#### MRI Secondary Outcomes

The MRI secondary outcomes included the occurrence of microvascular obstruction with late gadolinium enhancement on cardiac MRI 2 days after reperfusion (16), final infarct size at 6 months 16, 17, myocardial salvage 18, 19, 20, and myocardial salvage index 18, 19, 20 (both derived using final infarct size). Myocardial salvage (percentage of left ventricular volume) was defined as the difference between the initial jeopardized area at risk revealed by T2-weighted MRI at baseline 18, 19, 20 and final infarct size revealed by contrast-enhanced MRI at 6 months (18). The myocardial salvage index was defined as infarct size at 6 months indexed to the initial area at risk (19).

#### Safety Outcomes

The potential risks of the second catheterization (e.g., bleeding, contrast nephropathy) and procedure-related complications such as intraprocedural thrombotic events and health outcomes were included as safety outcomes.

Clinical outcome measures included the occurrence of heart failure, reinfarction, bleeding, and cardiac death during the index admission and after discharge, as defined in the Clinical Event Committee Charter (Online Appendix). Myocardial infarction and reinfarction were defined according to the Universal Definition of Myocardial infarction (21). All potential clinical events were adjudicated blinded to treatment allocation by an independent clinical event committee comprising 3 cardiologists who were not affiliated with our hospital (Online Appendix).

All study participants were followed for a minimum of 8 months after discharge. Information on adverse events was obtained by clinical review of the patients and primary and secondary care records during follow-up.

### Coronary angiogram acquisition and analyses

Coronary angiograms were acquired during usual care with cardiac catheter laboratory x-ray (Innova, GE Healthcare, Fairfield, Connecticut) and information technology equipment (Centricity, GE Healthcare). The angiograms underwent independent analysis in the Cardiovascular Research Foundation Angiographic Core Laboratory in New York, New York, by staff blinded to treatment assignment (Online Appendix).

### ECG and MRI acquisition and analyses

The ECGs underwent independent blinded analysis by an observer trained in the Glasgow ECG Core Laboratory (Glasgow, United Kingdom) (Online Appendix). Cardiac MRI was performed ∼2 days after reperfusion on a Siemens MAGNETOM Avanto (Erlangen, Germany) 1.5-T scanner with a 12-element phased array cardiac surface coil. The imaging protocol included cine MRI with steady-state free precession, T2-weighted edema imaging, and early and late gadolinium enhancement imaging (Online Appendix) 16, 17, 18, 19, 20. The initial area at risk was delineated with T2-weighted MRI (20). Microvascular obstruction was defined as a central dark zone on early contrast enhancement imaging 1, 3, 5, and 7 min post-contrast injection and present within an area of late gadolinium enhancement (16). Myocardial infarction was imaged using a segmented phase-sensitive inversion recovery turbo fast low-angle shot (17) radiofrequency pulse sequence 15 min after intravenous injection of 0.15 mmol/kg of gadoterate meglumine (Dotarem [gadoterate meglumine], Guebert S.A., Villepinte, France). The MRI scans were analyzed by observers blinded to the treatment group allocation of the study participants.

### Sample size

Based on a clinical audit in our hospital and a literature review 4, 5, 6, we estimated that the incidence of no-/slow-reflow (TIMI flow grade ≤2) during primary PCI would be 40% in selected patients with ≥1 of our predefined risk factors and 10% in the deferred PCI group. A minimum of 84 patients (42 per group) would provide 85% power to reject the null hypothesis with a type I error of 0.05.

### Statistical methods

Mean ± SD were used to summarize approximately normally distributed continuous data. Median (interquartile range [IQR]) or geometric mean ± SD were used to describe skewed continuous data. Number and percentage were used to summarize categorical data. All tests were 2-tailed and assessed at the 5% significance level. Comparisons of continuous variables used paired or unpaired *t* tests for normally distributed data or the Wilcoxon or *t* test after logarithmic transformation for skewed data. Differences in proportions were assessed using the Fisher exact or McNemar test for paired comparisons. Odds ratio and 95% confidence interval for treatment effects outcomes were assessed using exact logistic regression for binary outcomes and ordinal logistic regression for ordinal outcomes. All statistical analyses were performed using R version 2.15.2 (R Project for Statistical Computing, Vienna, Austria) or SAS version 9.2 (SAS Institute, Cary, North Carolina) (or higher versions of these programs).

The Robertson Center for Biostatistics acted as an independent coordinating center for randomization and its statisticians conducted the analyses. The study was monitored for safety by the sponsor. All serious adverse events were prospectively reported to the Pharmacovigilance Unit of the Clinical Trials Unit. The trial was approved by the National Research Ethics Service (reference 10/S0703-28). The registry was approved by the hospital’s Caldicott Guardian and Clinical Governance office. The trial sponsor is the National Waiting Times Center Board, NHS Scotland.

## Results

**Figure 1:**
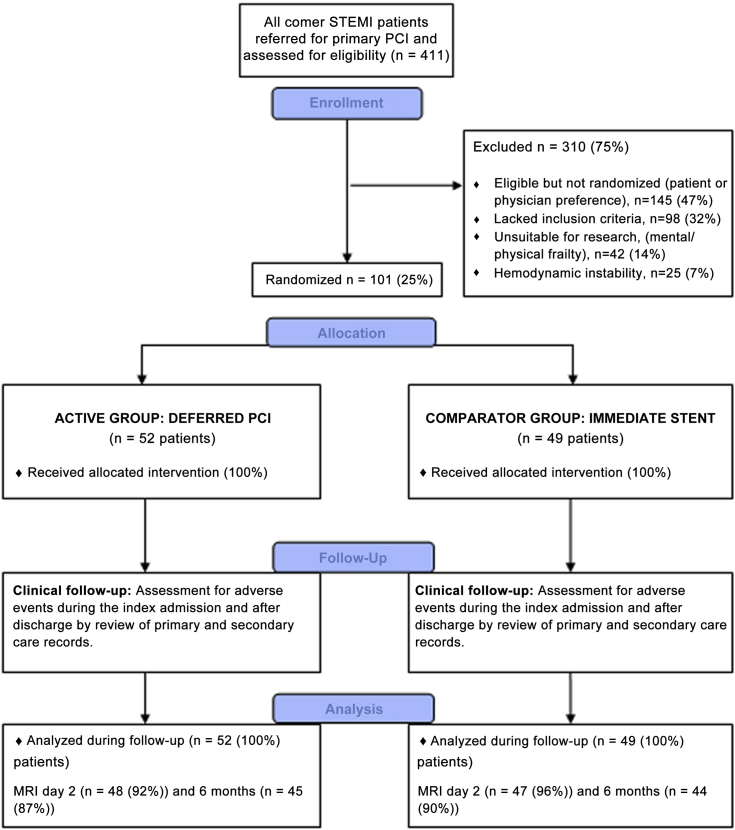
Study Flow Diagram MRI = magnetic resonance imaging; PCI = percutaneous coronary intervention; STEMI = ST-segment elevation myocardial infarction.

A total of 411 patients were treated with primary PCI between March 11, 2012 and November 21, 2012, and all of these patients were included in a registry (Fig. 1, Table 1). Of these, 101 patients (mean age, 60 years; 69% male) were randomized (52 in the deferred stenting group, 49 in the immediate stenting group (Fig. 1) by 8 of 13 cardiologists (62%). The trial stopped when all patients had a minimal follow-up period of 6 months, and all randomized patients were included in the analysis.

**Table 1:** Baseline Clinical and Angiographic Characteristics of All-Comers

Characteristics	Randomly Assigned Groups		Registry∗ (N = 310)
	Immediate Stenting (n = 49)	Deferred Stenting (n = 52)	
Clinical			
Age, yrs	61.7 ± 12.2	57.6 ± 10.9	61.4 ± 12.9
Male	36 (73.5)	34 (65.4)	196 (63.2)
Heart rate, beats/min	83 ± 17	77 ± 17	83 ± 32
Systolic blood pressure, mm Hg	138 ± 27	141 ± 24	131 ± 28∗
Diastolic blood pressure, mm Hg	79 ± 17	83 ± 11	77 ± 16∗
Diabetes mellitus,†	6 (12.2)	7 (13.5)	30 (9.7)
Previous MI	2 (4.1)	5 (9.6)	30 (9.7)
Previous PCI	2 (4.1)	2 (3.8)	21 (6.8)
Heart failure, Killip class at presentation†			
I	35 (71.4)	38 (73.1)	—
II	13 (26.6)	12 (23.1)	—
III	1 (2.0)	2 (3.8)	—
Procedure			
Time from symptom onset to reperfusion (first balloon or aspiration thrombectomy), min	183 (131–337)	166 (124–276)	184 (124–338)
Time from symptom onset to reperfusion >12 h	5 (10.2)	1 (1.9)	6 (5.9)
Coronary angiography			
No. of diseased arteries			
1	26 (55.3)	22 (45.8)	—
2	15 (31.9)	17 (35.4)	—
3	6 (12.8)	9 (18.8)	—
Culprit artery			
LAD	18 (36.7)	15 (28.8)	131 (42.3)∗
LCX	6 (12.2)	11 (21.2)	42 (13.5)∗
RCA	25 (51.1)	25 (48.1)	132 (42.6)
Vein graft	0 (0.0)	1 (1.9)	2 (0.6)
Left main	0 (0.0)	0 (0.0)	3 (1.0)
TIMI coronary flow grade pre-PCI‡			
0/1	39 (79.6)	40 (76.9)	200 (64.5)∗
2	7 (14.3)	6 (11.5)	43 (13.9)
3	3 (6.1)	6 (11.5)	67 (21.6)∗
Lesion length, mm∗	15.4 (11.2–20.6)	13.5 (11.2–17.8)	—
Coronary artery diameter at the start of the procedure, mm∗			
Proximal to the culprit lesion	3.2 ± 0.7	3.2 ± 0.6	—
Distal to the culprit lesion	2.7 ± 0.6	2.7 ± 0.6	—
Thrombus present,∗	47 (95.9)	51 (98.1)	284 (91.6)
Thrombus area, mm^2^∗	13.0 (8.3–20.2)	19.9 (12.0–1.3)	—
TIMI thrombus grade			
0/1	21 (42.9)	22 (42.3)	151 (48.9)
2	6 (12.2)	6 (11.5)	62 (20.1)
3	10 (20.4)	7 (13.5)	60 (19.4)
4	12 (24.5)	17 (32.7)	36 (11.7)
Jeopardized myocardium by the ECG Aldrich score (% left ventricle)§(14)	20 (17–30)	19 (15–26)	—
Procedure details			
Aspiration thrombectomy	42 (85.7)	46 (88.5)	—
Glycoprotein IIb/IIIa inhibitor therapy	46 (98.9)	51 (98.1)	—
Pre-dilation	36 (73.5)	46 (88.5)	—
Post-dilation	35 (71.4)	30 (57.7)	—
Final inflation pressure, kPa	17.4 ± 2.4	16.4 ± 3.2	—
Intracoronary adenosine therapy	4 (8.2)	3 (5.8)	—
No. of stents			
0	0	3 (5.8)	—
1	39 (79.6)	33 (63.5)	—
2	9 (18.4)	16 (30.8)	—
3	1 (2.0)	0	—
Contrast volume, ml	205 (172–250)	278 (238–312)	—

Values shown are n (%), mean ± SD, median (interquartile range) .

ECG = electrocardiogram; LAD = left anterior descending artery; LCA = left circumflex artery; MI = myocardial infarction; PCI = percutaneous coronary intervention; TIMI = Thrombolysis In Myocardial Infarction grade.

∗ The following clinical characteristics differed between the registry patients and the randomly assigned patients who were enrolled in the trial: systolic blood pressure (p = 0.003), diastolic blood pressure (p = 0.022), TIMI thrombus grade 4 (p < 0.0001), and TIMI flow grade pre-PCI (TIMI flow grade 0/1, p = 0.015; TIMI flow grade 3, p = 0.007). Quantitative coronary and electrocardiographic analyses were done in the randomized patients but not in the registry patients.

† Diabetes mellitus was defined as a history of diet-controlled or treated diabetes. Killip classification of heart failure after acute myocardial infarction: class I, no heart failure; class II, pulmonary rales or crepitations, a third heart sound, and increased jugular venous pressure; class III, acute pulmonary edema; class IV, cardiogenic shock. A diseased artery was defined as an epicardial artery (≥2 mm) with ≥1 lesions ≥50% of the reference vessel diameter.

‡ TIMI coronary flow grade pre-PCI was not assessable in 1 patient in the immediate stenting group. Intracoronary adenosine (10 to 30 μg) was administered as bolus therapy during primary PCI as clinically indicated for reduced coronary flow. The clinical and treatment characteristics of the patients included in the immediate stenting group and the deferred stenting group were similar except for the total volume of contrast, which was greater in the deferred stenting group (p < 0.0001). Procedure details and outcomes include the first and second procedures in the deferred stenting group. Two deferred stenting patients experienced culprit artery reocclusion before the planned second procedure. The coronary flow grades at the end of the first procedure and at the start of the second procedure differed in 3 other deferred stenting patients as follows: 2 patients changed from TIMI flow grade 3 to 2, and 1 patient changed from TIMI flow grade 2 to 3. None of the patients received bail-out or covered stents.

§ ST-segment elevation was measured on the baseline ECG before reperfusion to estimate the extent of initial myocardial jeopardy with the Aldrich ST-segment elevation score (14).

### Immediate versus deferred stenting groups

#### Angiographic Findings

The incidence of no-/slow-reflow after stenting (primary endpoint) was significantly lower in the deferred stenting group (odds ratio: 0.16; 95% confidence interval: 0.04 to 0.59; p = 0.006) (Table 1). Distal embolization and intraprocedural thrombotic events were also less frequent in the deferred stenting group (Table 2). After stenting, TIMI flow grade 3 and myocardial blush grade were higher in the deferred stenting group (Table 2). Within the deferred stenting group, there was a significant reduction in the proportion of patients with angiographic evidence of thrombus at the start of the second versus the first procedure (98.1% vs. 62.7%; p < 0.0001). Coronary thrombus area reduced significantly between the end of the first and start of the second angiograms (geometric mean for the ratio of the thrombus areas: 0.67; 95% confidence interval: 0.53 to 0.85). Compared with the intended stent strategy at the end of the first procedure, there was a significant increase in maximal stent diameter (in millimeters) (3.0 [IQR: 3.0 to 3.5] versus 3.5 [IQR: 3.0 to 4.0]; p < 0.0001) and length (in millimeters) (28 [IQR: 18 to 32] vs. 28 [20 to 40]; p = 0.002) evaluated by the same operator in the second procedure (Online Appendix). Three deferred stenting patients did not receive a stent. In 1 patient, repeat arterial access was not possible because of peripheral arterial disease. In the other 2 patients, the culprit lesions had only minimal residual stenosis.

**Table 2:** Primary and Secondary Angiographic and Electrocardiographic Outcomes

Outcome	Randomly Assigned Groups		Odds Ratio (95% CI)	p Value†	Registry (N = 310)
	Immediate Stenting (n = 49)	Deferred Stenting (n = 51)∗			
Primary outcome					
No- or slow-reflow (TIMI 0 to 2)‡					
Yes	14 (28.6)	3 (5.9)	0.16 (0.03–0.63)	0.005	45 (14.5)
Secondary angiographic outcomes					
No-reflow (TIMI grade 0 or 1)					
Yes	7 (14.3)	1 (2.0)	0.12 (0.03–1.02)	0.052	16 (5.2)
Final TIMI coronary flow grade post-PCI§					
3	39 (79.6)	50 (98.0)			273 (88.6)
2	6 (12.2)	0 (0.0)	0.08 (0.01–0.65)	0.018	25 (8.1)
0/1	4 (8.2)	1 (2.0)			10 (3.2)
Final TIMI myocardial blush grade post-PCI‖					
Missing	0	1			
3	26 (53.1)	40 (80.0)			
2	18 (36.7)	9 (18.0)	0.28 (0.11–0.65)	0.004	—
0/1	5 (10.2)	1 (2.0)			
No- or slow-reflow (TIMI grades 0–2), with MBG ≤1					
Missing	0	1			
Yes	5 (10.2)	1 (2.0)	0.18 (0.00–1.72)	0.195	
No- or slow-reflow (TIMI grades 0–2), with MBG ≤2					
Missing	0	1			
Yes	12 (24.5)	2 (4.0)	0.13 (0.01–0.64)	0.007	
All intraprocedural thrombotic events	28	9	—	—	68
Patients with at least 1 intraprocedural thrombotic event	16 (32.7)	5 (9.8)	0.23 (0.06–0.73)	0.010	63 (20.3)
Distal embolization	10 (20.4)	1 (2.0)	0.08 (0.02–0.60)	0.006	5 (1.3)
Other secondary outcome					
ECG: resolution of ST-segment elevation 60 min post-PCI					—
Complete, ≥70%	19 (38.8)	26 (50.0)			
Partial, 30% to <70%	21 (42.9)	15 (28.8)	0.77 (0.37–1.6)	0.484	
None, ≤30%	9 (18.4)	11 (21.2)			

Values are n (%). At the end of the final PCI, the percentage of diameter stenosis, final stent diameter, reference vessel diameter ratio, and corrected TIMI frame count were similar in both groups. In the deferred group, TIMI coronary flow grade, reference vessel diameters, final corrected TIMI frame count, and myocardial blush grade at the start of the second procedure compared with the end of the first procedure were similar in both groups.

CI = confidence interval; MBG = myocardial blush grade; other abbreviations as in Table 1.

∗ One of the patients in the deferred group did not have a second procedure because of failed vascular access; therefore, data from 51 participants in the deferred group have been included in the intention-to-treat analysis.

† The p value is the comparison between the immediate stenting group and the deferred group. Compared with the immediate stenting group, a lower proportion of patients in the registry group experienced no-/slow-reflow (45 [14.5%] vs. 14 [28.6%]; p = 0.01).

‡ No- or slow-reflow was assessed at any time during or at the end of PCI.

§ TIMI coronary flow grade was assessed post-PCI, at the end of the procedure, and was not assessable in 2 patients in the deferred group. The odds ratios for coronary flow grade post-PCI are the odds ratio for achieving a lower score in the deferred group relative to the immediate stenting group. The odds ratio calculations are described in the Methods section.

‖ TIMI MBG was assessed post-PCI, at the end of the procedure, and was not assessable in 2 patients in the deferred group. The odds ratios for TIMI MBG post-PCI are the odds ratio for achieving a lower score in the deferred group relative to the immediate stenting group. Corrected TIMI frame count was not assessable in 9 patients in the immediate stenting group and in 3 patients in the deferred group (data not shown).

#### MRI Findings

**Figure 2:**
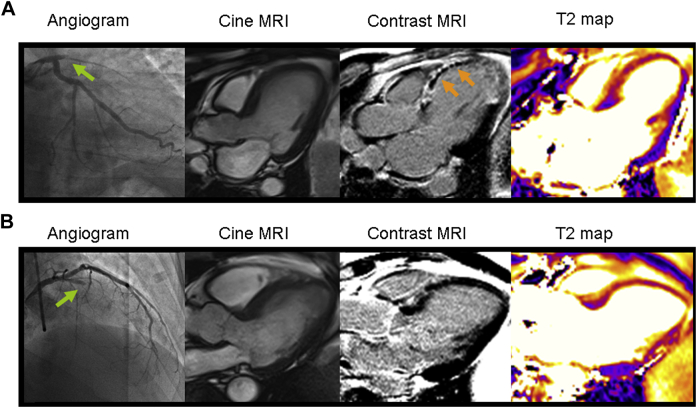
Two Patients With Acute Anterior STEMI One patient was treated by conventional primary PCI with immediate stenting and the other was treated with initial aspiration thrombectomy and then deferred stenting. Each patient had similar ischemic times (147 min and 163 min), and both were treated with similar antithrombotic therapies including 300 mg aspirin, 600 mg clopidogrel, 5,000 IU heparin, and intravenous tirofiban. **(A)** Usual care with immediate stenting. The angiogram **(left)** revealed proximal occlusion of the left anterior descending artery (**green arrow**, Thrombolysis In Myocardial Infarction [TIMI] grade 0 flow). Primary PCI was complicated by no-reflow (TIMI grade 1 flow). Two days later, cardiac MRI was performed. Cine MRI **(middle left)** revealed an extensive anteroapical left ventricular wall motion abnormality. Matched diastolic phase images obtained using late gadolinium enhancement imaging **(middle right)** revealed a transmural infarction with microvascular obstruction **(orange arrows)**. The area at risk revealed by T2-weighted imaging **(right)** was 42.2% of left ventricular mass, and the acute infarct size revealed by late gadolinium enhancement **(middle right)** was 39.6%. Microvascular obstruction depicted as the central dark zone within the infarct territory was 8.2% of the left ventricular mass. The left ventricular ejection fraction and end-systolic volume were 43.7% and 52.3 ml/m^2^, respectively. Six-month follow-up MRI revealed that the final infarct size was 32.1% of the left ventricular mass, and therefore myocardial salvage was 10.1%. **(B)** Deferred PCI. The angiogram **(left)** revealed a proximal occlusion of the left anterior descending artery (**green arrow**, TIMI grade 0 flow). Primary PCI in the left anterior descending coronary artery with deferred stent implantation was uncomplicated. Two days later, cardiac MRI was performed. Cine MRI **(middle left)** revealed an extensive anteroapical left ventricular wall motion abnormality. Despite similar ischemic times in both patients, this patient had minimal evidence of infarction on late gadolinium enhancement imaging **(middle right)** and no microvascular obstruction. The acute infarct size was 8.4% of the left ventricular mass. The area at risk **(right)** was similar between cases, at 43.1%, as was the left ventricular ejection fraction and normalized end-systolic volume, measured at 43.5% and 57.1 ml/m^2^, respectively, consistent with stunned but viable myocardium. Six-month follow-up MRI revealed that the final infarct size was 1.7% of the left ventricular mass, and therefore myocardial salvage was 41.4%.

The MRI results 2 days and 6 months post-MI are described in Table 3. Compared with immediate stenting, myocardial salvage (percentage of left ventricular mass) (19.7% [IQR: 13.8% to 26.0%] vs. 14.7% [IQR: 8.1% to 23.2%]; p = 0.027) and salvage index (68% (IQR: 54% to 82%) vs. 56% (IQR: 31% to 72%); p = 0.031) at 6 months were greater in the deferred stenting group (Fig. 2).

**Table 3:** Contrast-Enhanced Cardiac MRI Findings During Index Hospitalization and After 6-Month Follow-Up

	Immediate Stenting	Deferred PCI∗	p Value
MRI 2 days post-MI	n = 47	n = 48	
Microvascular obstruction	29 (61.7)	23 (47.9)	0.155
MRI 6 months post-MI	n = 44	n = 45	
Myocardial salvage, % of left ventricular mass†	14.7 (8.1–23.2)	19.7 (13.8–26.0)	0.027
Myocardial salvage index, %	56 (31–72)	68 (54–82)	0.031
Infarct size, % of left ventricular mass	14.3 (6.3–20.3)	9.0 (4.3–16.0)	0.181

Values are n (%) or median (interquartile range). The initial area at risk (percentage of left ventricular volume) revealed by MRI 2 days after MI was similar in patients randomized to immediate stenting (31.6 [IQR: 20.8 to 37.4]) compared with patients randomized to deferred PCI (28.4 [IQR: 23.4 to 36.6]; p = 0.577).

MRI = magnetic resonance imaging; other abbreviations as in Table 1.

∗ Compared with the immediate stenting group, favorable directional changes were observed in the deferred PCI group for left ventricular end-systolic volume, left ventricular end-diastolic volume, and ejection fraction (their changes at 6 months from baseline [data not shown]).

† When considering the degree of myocardial salvage according to specific patient characteristics (inclusion criteria), myocardial salvage (percentage of left ventricular mass) was significantly higher in the deferred stenting group compared with the immediate stenting group in patients with persistent ST-segment elevation on the electrocardiogram post-reperfusion and patients presenting with an occluded artery (19.05 [IQR: 13.57 to 26.52] vs. 12.60 [IQR: 6.20 to 23.20]; p = 0.039 and 19.70 [IQR: 13.70 to 26.40]vs. 12.55 [IQR: 6.52 to 19.67]; p = 0.001, respectively). There was directional consistency with a favorable treatment effect on salvage for deferred stenting across all the inclusion criteria (except for lesion length). The time from randomization to MRI was 60 (IQR: 18 to 97) h and 55 (IQR: 22 to 90) h in the immediate stenting and deferred groups, respectively.

### Adverse events and safety

#### In-Hospital Events After Randomization

In the deferred stenting group, recurrent ST-segment elevation myocardial infarction before stenting occurred in 2 patients. One patient had a severe intramural dissection within the culprit lesion in the left anterior descending coronary artery associated with absent flow in a large diagonal side branch. Five hours after initial reperfusion, the patient experienced recurrent chest pain associated with anterior ST-segment re-elevation. Repeat coronary angiography was performed within 30 min and confirmed reocclusion of the culprit artery. The patient received a stent, and his subsequent clinical course was uncomplicated. A second patient who inadvertently had not received low molecular weight heparin therapy in the Coronary Care Unit experienced a reinfarction before stenting. This patient was treated with a stent within 30 min of symptom onset and had an uncomplicated clinical course. One additional patient experienced an abrupt culprit artery closure and intraprocedural thrombotic event due to a guidewire-related dissection.

There were no bleeding events or in-hospital deaths. There was a greater volume of contrast used in the deferred stenting group (278 ml (IQR: 238 ml to 312 ml) vs. 205 ml (IQR: 170 ml to 250 ml); p < 0.0001). No cases of contrast nephropathy occurred.

### Post-discharge events

The duration of follow-up was 352 ± 79 days from randomization. Three patients in the deferred stenting group and 1 patient in the immediate stenting group experienced a non-STEMI. Two additional patients in the immediate stenting group were hospitalized with unstable angina, 1 of whom was treated with PCI. The incidence of recurrent MI post-randomization was similar for the 2 groups. In the deferred stenting group, 2 patients experienced an acute reinfarction during the index admission, and 3 patients experienced a non-STEMI during follow-up, whereas in the usual care group, 1 patient experienced a non-STEMI during follow-up (p = 0.108).

There was 1 noncardiovascular death due to small cell lung carcinoma in the deferred stenting group. All of these events are described in more detail in the Online Appendix.

## Discussion

We implemented a novel strategy to prevent no-reflow in at-risk patients with STEMI undergoing primary PCI. A simple approach was adopted for treatment stratification and randomization by the cardiologist. We identified patients with initial evidence of successful reperfusion and with clinical risk factors for no-reflow, and from these patients, the study participants were randomized to immediate stenting or to an intention-to-stent strategy within 4 to 16 h, including prolonged antithrombotic therapy. The strategy of deferred stenting in primary PCI represents a radical change from standard care.

Our trial provided new knowledge on primary PCI. We observed that deferred completion of PCI in selected STEMI patients reduced no-reflow, distal embolization, and intraprocedural thrombotic complications compared with conventional treatment with immediate stenting. Final coronary flow grade and myocardial blush grade were also better in the deferred stenting group. Two patients in the deferred stenting group experienced early recurrent myocardial infarction before the second procedure. During longer term follow-up, myocardial salvage measured with cardiac MRI was significantly greater in the deferred stenting group. In other words, when indexed to the initial extent of jeopardized myocardium (i.e., the ischemic area at risk), final infarct size was smaller in the patients randomized to deferred PCI. The MRI results obtained after 6 months of follow-up indicate a beneficial treatment effect that was sustained over time. Because myocardial salvage is a prognostically validated therapeutic target in primary PCI 2, 9, the favorable long-term effect of deferred stenting on myocardial salvage is clinically relevant.

We also found that in the deferred PCI group, the approach to PCI differed at the second procedure compared with the first for the same operator. The maximal stent diameter in the second procedure was 0.5 mm greater and the range of stent length was greater. Three-fourths of patients had an increase in stent diameter. These observations indicate that vessel dimensions are greater at the second procedure compared with the first, in keeping with attenuation of coronary artery tone with time from reperfusion.

Our trial results reflect a balance of potential benefits and potential risks. The trial was conducted during usual care, and our intervention was based on simple clinical eligibility criteria. The antithrombotic strategy involved a mechanical component (i.e., deferral of stent implantation to avoid/minimize thrombus embolization) and a therapeutic component based on prolonged treatment with low molecular weight heparin (1 mg/kg) and glycoprotein IIb/IIIa inhibitor therapy during the interval between the first and second PCI procedures. Glycoprotein IIb/IIIa inhibitor therapy is an evidence-based antithrombotic treatment 2, 9 and was included in therapeutic strategy to reduce thrombus burden before stent implantation in the deferred stenting group (9). Although these treatments also increase the risk of bleeding, no bleeding problems occurred in the deferred stenting group, probably because radial artery access was used in all patients. Accordingly, our strategy has the potential to be widely applicable.

Our strategy was based on selection of patients with at least 1 clinical and/or angiographic risk factor for no-reflow 4, 5, 6. We believed that the intervention would not be appropriate in “all-comers” for 3 reasons. First, the efficacy of deferred stenting was likely to be greatest in the patients at highest risk of no-/slow-reflow. Second, the risk of recurrent myocardial infarction could not be mitigated in patients who were at low risk of no-reflow on clinical grounds. Third, a strategy that involved all-comers would be difficult to implement due to the large number of additional second procedures. To assess whether clinicians could stratify patients at risk of no-reflow, information on all-comers was collected, and those who were not randomized were included in a registry.

The clinical risk profiles of the randomized and registry patients differed. Compared with the registry patients, TIMI thrombus grade 4 and an occluded culprit artery (TIMI flow grade 0/1) were much more common in the trial patients. Greater thrombus burden and an occluded culprit artery are both associated with large infarct size (22) and an adverse prognosis 2, 9. These baseline differences between randomized and registry patients can be explained by appropriate risk stratification and patient selection by the cardiologists at the time of primary PCI.

Compared with the randomized trial participants, the no-/slow-reflow rate of 14.5% in the registry was nearly half of the incidence of this event observed in the immediately stented patients and more than double the incidence of no- or slow-reflow in the deferred stenting group. This finding indicates that the patient selection approach correctly identified a subgroup of STEMI patients in whom the incidence of no-/slow-reflow was lower (Table 2). Furthermore, the incidence of no-/slow-reflow in the subgroup of registry patients who were eligible for inclusion but not randomized due to patient or physician preference (Fig. 1) was 23.4%, which was very similar to the rate observed in the immediate stenting group. Although the observed rate of no-reflow in the immediate stenting group was lower than predicted, we still observed a significant reduction in the deferred stenting group.

Our study was performed during normal emergency care. However, as might be expected with a new intervention that represents a radical change from standard care, patient enrollment was influenced by physician preference, and in the absence of clinical evidence to support this strategy, 5 of 13 cardiologists in our primary PCI service did not randomize any patients.

Thrombus is mechanistically involved in no-reflow, and stent implantation may cause distal embolization of clot and microvascular thrombosis (23). Based on the rationale for our intervention, we examined whether coronary thrombus burden might be lower at the start of the second PCI compared with the start of the first procedure (when stenting is normally performed), and this indeed was the case. Furthermore, thrombus in the culprit artery had dissipated during the intervening period. Thus, coronary stent implantation in the deferred stenting group of patients occurred when thrombus burden was less, and so the substrate for distal embolization and microvascular thrombosis had diminished. This may explain the lower incidence of no-reflow in the deferred stenting group.

Two patients in the deferred stenting group had early recurrent myocardial infarction. One of these patients had a complex culprit lesion with an intramural dissection (24) and persistently reduced side-branch flow (TIMI grade 1). The other patient was a protocol violation as he had not received low molecular weight heparin after the initial procedure. Both patients were treated with PCI expeditiously and without complication. These events contain learning that should be used to optimize the design of a future clinical trial. For example, persistent flow reduction (TIMI grade 0/1) in the side branch of a culprit bifurcation lesion would be an exclusion criterion. Overall, the balance of the benefit of reduced no-reflow versus the risk of recurrent myocardial infarction needs to be tested in a large multicenter, randomized, controlled trial.

Therapeutic strategies for the prevention and treatment of no-reflow have not improved clinical outcomes in large multicenter randomized trials 2, 8, 25, 26, 27. Other clinical trials with different approaches to deferred stenting in primary PCI include MIMI (Minimal Invasive Procedure for Myocardial Infarction [NCT01360242]), PRIMACY (Primary Reperfusion Secondary Stenting Trial [NCT01542385]), and DANAMI-3 (DANish Study of Optimal Acute Treatment of Patients With ST-elevation Myocardial Infarction [NCT01435408]). Recent studies 28, 29, 30, 31, including 3 nonrandomized case series 28, 29, 30 and a systematic review (31), support the notion that deferred stenting may be safe in appropriately selected STEMI patients.

### Implications for clinical practice

Our strategy of deferred stenting in selected STEMI patients with risk factors for no-reflow represents a potential new treatment paradigm. The strategy involves a balance between competing risks and benefits that merits prospective evaluation in a large clinical trial. On the one hand, we have shown that deferred stenting reduces no-reflow and increases myocardial salvage. On the other hand, there may be an increased risk of early recurrent STEMI. Based on our experience, the design of the intervention should be adapted to further mitigate the risk of early coronary reocclusion (e.g., a patient with side-branch occlusion would not be suitable). A deferred stent strategy involves a second procedure, and so procedure-related costs may be higher. Our study design timed the second procedure 4 to 16 h after the first to keep the second procedure within working hours and thus optimize feasibility. On the other hand, the strategy has the potential to reduce healthcare costs overall by reducing the clinical consequences of no-reflow (e.g., heart failure and its related cost burden). Only a large clinical trial designed to assess patient experience, health outcomes, quality of life, and cost-effectiveness can address these uncertainties.

### Study limitations

Investigators and patients were unblinded in our study. For this reason, the primary and secondary outcomes underwent independent analysis blind to treatment group assignment to prevent ascertainment bias. Our initial estimates for the expected incidences of no-/slow-reflow were slightly higher than the observed rates. The reasons for this may be multifactorial and may reflect the effect of core laboratory adjudication over investigator-reported events. Our study design did not include an angiographic control in the immediate stenting group, but we do not think that this is relevant because the occurrence of no-reflow and other angiographic sequelae, such as intraprocedural thrombotic events, is due to the effect of PCI, so additional invasive angiography in the control group seems unnecessary. The minimal delay of 4 h to the second procedure in the deferred stenting group was relatively short and may not have been sufficient time to permit significant reduction in thrombus size. Although 2 patients experienced recurrent STEMI, their outcome was favorable. The rate of recurrent ischemia in the deferred stenting group was low, and, considering the limited sample size, we cannot exclude the possibility that this was due to chance. TIMI frame count was not assessable in several of the angiograms. Because deferred stenting involves a second invasive procedure with radiographic contrast medium, on safety grounds, radial artery access is recommended and advanced peripheral vascular disease and severe renal dysfunction (e.g., glomerular filtration rate <30 ml/min) should be exclusion criteria. Glycoprotein IIb/IIIa inhibitor therapy and unfractionated heparin were used rather than bivalirudin (2), and because the former antithrombotic combination therapy almost certainly remains the most common combination used worldwide, we think that our results are generalizable. Our standard-care antiplatelet strategy involved oral clopidogrel given at the time of the first medical contact (2). Because more effective antiplatelet drug therapies are available, such as prasugrel, ticagrelor, and cangrelor (2), we postulate that the efficacy and safety of the deferred strategy could be further enhanced with one of these drugs instead of clopidogrel. Some of the clinical characteristics (e.g., time from symptom onset to reperfusion >12 h) and outcomes (e.g., some MRI and electrocardiographic parameters) were numerically, but not statistically, different between treatment groups, which may reflect the limited sample size.

## Conclusions

For the first time, we conducted a proof-of-concept trial and found that deferred stenting in primary PCI reduced no-reflow and increased myocardial salvage compared with conventional primary PCI with immediate stenting. Two patients had recurrent myocardial infarction, which represents important balancing information on potential risks. The strategy is simple, pragmatic, and potentially widely applicable. Our results support the rationale for a substantive multicenter clinical trial to assess the cost-effectiveness of early deferred completion of PCI after reperfusion versus conventional treatment in STEMI patients at risk of no-reflow.
