# Supplementary material for: A Randomized Trial of Deferred Stenting Versus Immediate Stenting to Prevent No- or Slow-Reflow in Acute ST-Segment Elevation Myocardial Infarction (DEFER-STEMI)
Source: J Am Coll Cardiol. 2014 May 27;63(20):2088–98. doi: 10.1016/j.jacc.2014.02.530 (PMC4029071; doi:10.1016/j.jacc.2014.02.530)
Supplement: Online Data [file mmc1.docx]

# Supplementary data file

## A Randomized, Controlled Trial of Deferred Stenting Versus Immediate Stenting to Prevent No-Reflow in Acute ST-Segment Elevation Myocardial Infarction NCT01717573

## Adverse events after discharge

The mean (SD) duration of follow-up was 173 (80) days from randomization. Three patients in the deferred group and one patient in the immediate stenting group experienced a non–ST- segment elevation myocardial infarction. Two patients in the immediate stenting group were hospitalized with unstable angina, one of whom was treated with PCI.

The patients in the deferred group who had a recurrent non–ST-segment elevation myocardial infarction presented at 4, 11, and 17 weeks post-randomization. In the first two patients, coronary angiography revealed unobstructed coronary arteries and both patients were treated with medical therapy. The third patient who presented at 17 weeks with an inferolateral non–ST-segment elevation myocardial infarction had originally not received a coronary stent during the index procedures. The initial primary PCI had involved balloon angioplasty to a culprit lesion in the left circumflex coronary artery. However, the subsequent deferred procedure 8 hours later was unsuccessful because peripheral arterial disease prevented repeat arterial access. This patient's in-hospital course was uncomplicated and she was discharged. When repeat coronary angiography was performed 17 weeks later, there was normal blood flow in the circumflex artery but evidence of organized thrombus at the site of the culprit lesion. The patient received a bare metal stent and had an uncomplicated recovery.

One patient in the immediate stenting group had a non-ST elevation myocardial infarction 16 weeks after randomization. Coronary angiography at that time revealed unobstructed coronary arteries and the patient was treated with medical therapy. Two other patients had unstable angina and one was treated with PCI.

No bleeding events occurred in either group. One non-cardiovascular death occurred due to small cell lung carcinoma. The patient had been randomized to the deferred group and died 13 months after randomization.

**Comparison of stent strategy between procedures in the deferred group**

Compared with the intended stent strategy at the end of the first procedure, there was a 0.5-mm increase in maximum stent diameter (p < 0.0001) and 3-mm increase in total length (p = 0.002), evaluated by the same operator for both procedures (Table). Three patients who did not receive a stent in the second procedure were excluded from the analysis.

|  | Deferred stenting group  n = 49 | |  |
| --- | --- | --- | --- |
| Characteristic | Procedure 1 | Procedure 2 | p value |
| Maximum stent diameter, mm | 3.0 (3.0, 3.5) | 3.5 (3.0, 4.0) | <0.0001 |
| Total stent length, mm | 28 (18, 32) | 28 (20, 40) | 0.002 |
| Patients with an increase in maximum stent diameter for that procedure, n (%) | 2 (4%) | 36 (75%) |  |

| Characteristic |  | p value |
| --- | --- | --- |
| Median increase in maximum stent diameter in procedure 2 versus procedure 1, mm | 0.5 | <0.0001 |
| Median increase in total stent length in procedure 2 versus procedure 3, mm | 3 | 0.002 |
