# Supplementary material for: A Randomized Trial of Deferred Stenting Versus Immediate Stenting to Prevent No- or Slow-Reflow in Acute ST-Segment Elevation Myocardial Infarction (DEFER-STEMI)
Source: J Am Coll Cardiol. 2014 May 27;63(20):2088–98. doi: 10.1016/j.jacc.2014.02.530 (PMC4029071; doi:10.1016/j.jacc.2014.02.530)
Supplement: Online Data [file mmc2.docx]

# Supplementary Methods Including Clinical Event Committee (CEC) Adjudication Charter

## A Randomized, Controlled Trial of Deferred Stenting Versus Immediate Stenting to Prevent No-Reflow in Acute ST-Segment Elevation Myocardial Infarction

## NCT01717573

# Table of contents

[Supplementary Methods including Clinical Event Committee (CEC) Adjudication Charter 1](#_Toc373963363)

[Setting 3](#_Toc373963364)

[Coronary angiogram acquisition and analyses 3](#_Toc373963365)

[Percutaneous coronary intervention 3](#_Toc373963366)

[Usual care and deferred PCI 4](#_Toc373963367)

[Angiographic core laboratory analysis 5](#_Toc373963368)

[Outcome definitions 5](#_Toc373963369)

[ECG analysis 8](#_Toc373963370)

[MRI acquisition and analyses 9](#_Toc373963371)

[Biochemical assessment of infarct size 12](#_Toc373963372)

[Trial Management 12](#_Toc373963373)

[Definition of adverse events 12](#_Toc373963374)

[Clinical Event Committee (CEC) Adjudication Charter 14](#_Toc373963375)

[Rationale for a clinical event adjudication committee 16](#_Toc373963376)

[Objective of the CEC Charter 17](#_Toc373963377)

[Composition and responsibilities of the CEC 18](#_Toc373963378)

[Events to be reviewed by the CEC 20](#_Toc373963379)

[Adverse Event definitions 22](#_Toc373963380)

[Adjudication process 45](#_Toc373963381)

[Clinical data to be provided 49](#_Toc373963382)

[CEC Quality assurance 53](#_Toc373963383)

[Appendix A: Adjudication Process Flowcharts 54](#_Toc373963384)

[References 56](#_Toc373963385)

# Setting

Screening, enrollment, and data collection were performed in the Golden Jubilee National Hospital, Glasgow, United Kingdom.

# Coronary angiogram acquisition and analyses

Coronary angiograms were acquired during usual care with cardiac catheter laboratory X-ray (Innova®) and IT equipment (Centricity®) made by GE Healthcare.

# Percutaneous coronary intervention

Consecutive STEMI admissions referred for primary PCI were screened for the inclusion and exclusion criteria. During ambulance transfer to the hospital, the patients received 300 mg of aspirin, 600 mg of clopidogrel and 5000 IU of unfractionated heparin.^2,9^ The initial primary PCI procedure was performed using radial artery access. A conventional approach to primary PCI was adopted in line with usual care in our hospital. ^2,9^ Conventional bare metal and drug eluting stents were used. Covered stents or investigational stents designed to reduce thrombus embolization were not used.^26^ The guideline to cardiologists recommended minimal intervention for initial reperfusion with aspiration thrombectomy only or minimal balloon angioplasty (e.g., a compliant balloon sized according to the reference vessel diameter and inflated at 4-6 atm 1-2 times. Bail-out PCI because of coronary dissection or repeated angioplasty to minimize stenosis severity was not permitted and patients treated in this way were not eligible to participate. Provided TIMI grade 3 flow had been achieved with initial reperfusion therapy, then the residual stenosis severity had no influence on eligibility. During PCI, glycoprotein IIbIIIa inhibitor therapy was initiated with high dose tirofiban (25 μg/kg/bolus) followed by an intravenous infusion of 0.15 μg/kg/min for 12 hours.^2,9^ No reflow was treated according to contemporary standards of care with intra-coronary nitrate (i.e., 200 μg) and adenosine (i.e., 30 – 60 μg)^2,9^, as clinically appropriate.

In patients with multivessel coronary disease, multivessel PCI was not recommended, in line with clinical guidelines. ^2,9^ The subsequent management of these patients was symptom guided.

# Usual care and deferred PCI

The deferred PCI strategy involved an intention-to-stent 4 to 16 hours after initial coronary reperfusion. A minimum of 4 hours was adopted to permit the beneficial effects of reperfusion and anti-thrombotic therapies to reduce thrombus burden. In practice, an 8 hour guideline was recommended for the deferred PCI to ensure that all patients could be treated between 0700 – 2300 hrs during the first 24 hours of admission to ensure that the second procedure occurred at a time which facilitated a rest period for the patient and the staff. Finally, an upper limit of 16 hours was set to avoid prolongation of the hospital admission.

The treatment protocol for deferred patients included transfer to the Coronary Care Unit, continuous intravenous infusion of glycoprotein IIbIIIa inhibitor therapy (tirofiban, 0.15 μg/kg/min) and administration of subcutaneous low molecular weight heparin (enoxaparin, 1 mg/kg 12 hourly) for up to 16 hours. The radial artery sheath used for PCI was retained or removed according to operator and patient preference. Arterial blood pressure and the radial sheath site were monitored in the Coronary Care Unit. All patients also had continuous ECG monitoring in the Coronary Care Unit. Usual care included immediate stenting in the catheter laboratory and intravenous glycoprotein IIbIIIa inhibitor therapy for 12 hours (tirofiban, 0.15 μg/kg/min). In both groups drug eluting or bare metal stents were used and post-dilatation performed according to operator judgement.^9^ After the PCI procedure was completed the patients returned to the Coronary Care Unit and were treated with optimal secondary prevention measures.^2^

# Angiographic core laboratory analysis

The coronary angiograms of study participants were coded, de-identified and analyzed at an independent core laboratory (Cardiovascular Research Foundation, New York, New York) by technicians blinded to randomization and clinical outcomes. Quantitative analyses of coronary, stent, and thrombus dimensions were performed with Medis QAngio XA v7.2.34 (Medis Medical Imaging Systems, Leiden, the Netherlands) image analysis software. In addition to routine pre- and post-procedural quantitative and qualitative assessments, additional analyses of every cine angiographic frame were performed. Intra-procedural complications were independently assessed for each angiographic run.

### Registry patients

The coronary angiograms of the non-randomized patients were analyzed by 3 experienced interventional cardiologists (M.B., A.S., W.S.H.) who were independent of the lead site. All of the angiograms were independently and separately adjudicated by two cardiologists (M.B., A.S.) and disagreements were resolved by consensus established independently by a third cardiologist (W.S.H.).

# Outcome definitions

Coronary blood flow can be described based on the visual assessment of coronary blood flow revealed by contrast injection into the coronary arteries^1^.

| TIMI Coronary Flow Grade |  |
| --- | --- |
| 0 | No flow |
| 1 | Minimal flow past obstruction |
| 2 | Slow (but complete) filling and slow clearance |
| 3 | Normal flow and clearance |

The TIMI flow grade is straightforward to evaluate in the catheter laboratory hence TIMI flow grade was used as an eligibility criterion for participation in the study.

Tissue myocardial perfusion (blush) grade

Coronary angiography also provides other information on coronary blood flow and myocardial perfusion. The TIMI blush grade is an ordinal score for contrast washout at the end of the angiogram^2^, and the TIMI blush grade is also predictive of prognosis^2^.

| TIMI Blush grade |  |
| --- | --- |
| 0 | No myocardial blush |
| 1 | Minimal blush and very slow clearing (e.g., present at beginning of next cine) |
| 2 | Good blush with slow clearing of myocardial contrast (present at end of cine but gone at beginning of next) |
| 3 | Good blush and normal clearing (i.e., gone by end of cine) |

## TIMI frame count

The TIMI frame count is a simple objective continuous variable index of coronary blood flow, representing the amount of time (in frames) for contrast dye to reach a standardized distal landmark, corrected for vessel length^3^. The corrected TIMI frame count (CTFC) is predictive of prognosis^3^.

**Method:** *Corrected TIMI frame count (normal < 27 frames).* The CTFC is the number of cine frames required for contrast to first reach standardized distal coronary landmarks in the culprit artery and was measured with a frame counter on a cine viewer. A frame count of 100, a value that is the 99^th^ percentile of patent vessels, was imputed to an occluded artery. CTFC is a measure of time, and data were converted when necessary according to film speed (e.g.,, 30 frames/s). The CTFC was divided by 30 to calculate the transit time for dye to traverse the length of the artery to the landmark in seconds and multiplied by 1000 to calculate the time in milliseconds. This was used along with the heart rate to calculate the fraction of a cardiac cycle required for dye to traverse the artery: fraction of cardiac cycle (CTFC/30 seconds) / (60s/heart rate). Calculation of the fraction of a cardiac cycle required for dye to traverse the culprit artery normalizes the CTFC for heart rate.

## Intra-procedural thrombotic events

An intra-procedural thrombotic event was defined as the development of new or increasing thrombus, abrupt vessel closure, no reflow or slow reflow, or distal embolization occurring at any time during the procedure^4^. Embolization was defined as a distal filling defect with an abrupt 'cutoff' in one of the peripheral coronary artery branches of the infarct-related vessel, distal to the site of angioplasty.^9^ Each complication was assessed relative to the status of the previous frames. Thus, if thrombus was present at baseline but then resolved only to recur later, this was coded as an intra-procedural thrombotic event. Similarly, thrombus at baseline that qualitatively “grew” in subsequent frames was considered an intra-procedural thrombotic event. Conversely, baseline thrombus that persisted in size without growing, diminished, or resolved was not considered an intra-procedural thrombotic event.

## Comparison of stent strategy between procedures for the deferred group

When possible, the intended stent strategy at the end of the first procedure was prospectively recorded and compared to the actual approach used at the second procedure, by the same operator. If this was not captured at the time of the first procedure, then the operator, who performed the second procedure, re-evaluated the first angiogram blinded to patient identity and made a treatment decision on maximum stent diameter and total stent length.

# ECG analysis

A 12 lead electrocardiogram (ECG) was obtained before coronary reperfusion and 60 minutes afterwards with Mac-Lab® technology (GE Healthcare) in the catheter laboratory and a MAC 5500 HD recorder (GE Healthcare) in the Coronary Care Unit. The ECGs were acquired by trained cardiology staff. The ECGs were de-identified and transferred to the local ECG management system. The ECGs were then analyzed by R.W. who was trained by the University of Glasgow ECG Core Laboratory which is certified to ISO 9001: 2008 standards as a UKAS Accredited Organization.

The Aldrich score was used to assess initial infarct size on the baseline ECG^5^.A 12-lead ECG was acquired at presentation prior to reperfusion and 60 minutes after initial coronary reperfusion and the ST-seg­ments on the post-procedural ECG were compared with those on the ECG at presentation. The degree of resolution of ST-segment elevation was cate­gorized as greater than 50% resolution or not^6,7^.

# MRI acquisition and analyses

Cardiac MRI was performed approximately 2 days post-MI on a Siemens MAGNETOM Avanto (Erlangen, Germany) 1·5-Tesla scanner with a 12-element phased array cardiac surface coil. The imaging protocol included cine MRI with steady state free precession, T2 weighted edema MRI and early and late gadolinium enhancement imaging^8^. Microvascular obstruction was defined as a central dark zone on early contrast enhancement imaging 1, 3, 5 and 7 minutes post-contrast injection and present within an area of late gadolinium enhancement^8-10^. Myocardial infarction was imaged using a segmented phase-sensitive inversion recovery turbo fast low-angle shot radiofrequency pulse sequence 15 minutes after intravenous injection of 0.15 mmol/kg of gadoterate meglumine (Gd2+-DOTA, Dotarem, Guebert S.A.). Typical imaging parameters were: matrix = 192 x 256, flip angle = 25°, TE = 3·36 ms, bandwidth = 130 Hz/pixel, echo spacing = 8·7ms and trigger pulse = 2. The voxel size was 1·8 x 1·3 x 8 mm^3^.

### MR image analyses

The images were analyzed on a Siemens workstation by three MRI trained clinicians (C.B, N.A., I.M.) with at least 2 years experience in patients with acute MI, including protocols with T2­ weighted MRI. These observers were blinded to the treatment group allocation of the study participants. The MRI analyses were coordinated by D.C. Left ventricular dimensions, volumes and ejection fraction were quantified using computer assisted planimetry (syngo MR®, Siemens Healthcare, Erlangen, Germany).

### Infarct definition and size

The presence of acute infarction was established with MRI based on abnormalities in cine wall motion, rest first-pass myocardial perfusion, and delayed-enhancement imaging. In addition, supporting changes on the ECG and coronary angiogram were also required. Acute infarction was considered present only if late gadolinium enhancement was confirmed on both the axial and long axis acquisitions. The myocardial mass of late gadolinium enhancement (grams) was quantified by a semiautomatic detection method using a signal intensity threshold of >5 SD above a remote reference region and expressed as a percentage of total left ventricular mass^8^.

### Microvascular obstruction

Microvascular obstruction regions were included within the infarct area. Microvascular obstruction was classified as present (central dark zone with a subendocardial or intramural distribution) or absent (dots or nil) and also expressed as a percentage of total left ventricular mass^8-10^.

### Area at risk

Since water content and mobility directly influence myocardial T2-relaxation time (T2), and myocardial edema closely represents the ischemic area-at-risk following acute MI,^18^ T2-mapping was used to delineate the myocardial area-at-risk.^20^ A T2 prepared TrueFISP pulse sequence was used to produce single-shot T2 prepared images, each with a different T2 preparation.^20^ The T2prepared TrueFISP images were acquired at intervals of at least 3 R-R intervals to allow for sufficient magnetization recovery in between acquisitions. Motion correction was enabled to prevent misregistration between images. T2 is estimated by pixel-wise fitting assuming a mono-exponential signal decay and a color-scaled motion-corrected myocardial T2 map was then generated.^20^

### Salvage

Myocardial salvage (% left ventricular volume) was defined as the difference between the initial jeopardized area-at-risk revealed by T2-weighted MRI and final infarct size revealed by contrast-enhanced MRI at 6 months^18^. The myocardial salvage index was defined as myocardial salvage indexed to the initial area-at-risk.^19^

## MR image analyses

The MR images were separately analyzed on a Siemens workstation by three clinicians (N.A, J.L., I.M.) with at least 2 years of CMR experience, respectively. These observers were independent of the clinical service and were blinded to treatment group assignment. Left ventricular dimensions and volumes and ejection fraction were quantified using computer assisted planimetry.

Quantitative assessments of the area-at-risk were performed by I.M. The approach to image analysis, including inter-observer agreement, has been previously reported.^18,19^ The jeopardized area-at-risk on each axial image was defined as the percentage of left ventricular area delineated by the hyperintense zone on T*_2_*-weighted images.^18,19^ Myocardial salvage was calculated by subtraction of percent final infarct size from percent area-at-risk.^18,19^ The myocardial salvage index was calculated by dividing myocardial salvage by the initial area-at-risk.

### Reference ranges

Reference ranges used in the laboratory were 105 – 215 g for LV mass in men, 70 – 170 g for LV mass in women, 77 – 195 ml for LV end-diastolic volume in men, 52 – 141 ml for LV end-diastolic volume in women, 19 – 72 ml for LV end-systolic volume in men and 13 – 51 ml for LV end-systolic volume in women.

# Biochemical assessment of infarct size

Troponin T was measured (Elecsys Troponin T, Roche) as a biochemical measure of infarct size. The high sensitive assay reaches a level of detection of 5 pg/ml and achieves less than 10% variation at 14 pg/ml corresponding to the 99th percentile of a reference population. A blood sample was routinely obtained 12 – 24 hours after hospital admission.

# **Trial Management**

The trial was conducted in line with Guidelines for Good Clinical Practice (GCP) in Clinical Trials^11^. Trial management included a Trial Management Group, an independent Clinical Event Committee (CEC) and an independent Clinical Trials Unit. Day to day study activity was coordinated by the Trial Management Group who was responsible to the Sponsor which was responsible for overall governance and that the trial was conducted according to GCP standards^11^.

Clinical events were assessed and validated by an independent CEC comprised of 3 consultant cardiologists from Ninewells Hospital, Dundee (Chair, Dr John Irving). The CEC followed an agreed charter and the CEC were blinded to randomization and treatment group assignment.

# Definition of adverse events

A comprehensive definition of adverse events and their adjudication is detailed in the Clinical Event Committee Charter.

1) Major Adverse Cardiovascular Events (MACE) is the composite of 'cardiovascular death, non-fatal MI, unplanned hospitalization for transient ischemic attack or stroke.'

2) 'Major Adverse Cardiac Events' are defined as 'cardiac death, or unplanned hospitalization for myocardial infarction or heart failure

Following initial coronary revascularization during the index hospitalization, subsequent percutaneous coronary intervention and coronary artery bypass graft procedures were classified as non-major adverse events.

3) Procedure-related myocardial infarction was defined according to the Third Universal Definition of Myocardial Infarction (Type 4 for percutaneous coronary intervention and Type 5 for coronary artery bypass graft)^12^.

4) Contrast-induced nephropathy: is defined as either a greater than 25% increase of serum creatinine or an absolute increase in serum creatinine of 0.5 mg/dl after a radiographic examination using a contrast agent.^13^

5) Bleeding: is defined according to the ACUITY criteria^14^: major bleed = intracranial or intraocular bleeding; bleeding at the site of angiography requiring intervention; a hematoma of 5 cm in diameter; a reduction in hemoglobin level of at least 4 g/dL in the absence of overt bleeding or 3 g/dL with a source of bleeding; or transfusion.

# Clinical Event Committee (CEC) Adjudication Charter

Title: A Randomized Controlled Trial to Assess Whether Deferred Stenting in Acute STEMI Patients Might Reduce the Incidence of No-Reflow Versus Conventional Treatment With Immediate Stenting

Version Date: November 2012

Clinical Trial Registration Number NCT 01717573

#

## CEC Adjudication Charter : Introduction

BACKGROUND: During primary PCI, stent deployment and post-dilatation are associated with no-reflow, which is associated with adverse outcomes such as myocardial infarction, heart failure and death. The mechanisms for no reflow include distal embolization of thrombus, enhanced thrombus formation and vascular spasm. No reflow is associated with risk factors such as prolonged duration of ischemia, heavy thrombus burden, persistent ST elevation and long stent length. The pathophysiology of reflow and ischemic heart injury classically involves microvascular obstruction (MVO). There are no evidence-based treatments to prevent or treat reflow which represents an unmet clinical problem. Since stenting and post-dilatation are causally associated with no reflow during emergency percutaneous coronary intervention (PCI) for ST elevation myocardial infarction, we reasoned that after initial mechanical coronary reperfusion, deferred stenting for a limited period of time might be associated with fewer episodes of no reflow compared to usual care with direct stenting.

ACTIVE HYPOTHESIS: Once normal antegrade flow has been re-established with initial aspiration thrombectomy and/or balloon angioplasty at the beginning of primary PCI, compared with usual care with direct stenting, a strategy of deferred stenting for 4 -16 hours to permit the beneficial effects of normalized coronary blood flow and anti-thrombotic therapies will reduce the incidence of no reflow in at-risk STEMI patients.

DESIGN: In consecutive STEMI patients with risk factors for no reflow and who have given informed consent, when normal flow has been established (TIMI 3) by initial aspiration thrombectomy and/or balloon angioplasty, participants will be randomized to deferred stenting or usual care with direct stenting. All patients will receive dual anti-platelet therapy. Patients who are randomized to deferred stenting will receive intravenous glycoprotein IIbIIIa inhibitor and anti-coagulation with low molecular weight heparin. Patients who are screened and not eligible to be randomized will be prospectively entered into a registry. Study assessments for feasibility, safety and efficacy will be prospectively performed. An independent clinical event committee will review all serious adverse events. Study endpoints will be subject to core laboratory analyses. Should our hypotheses for safety, efficacy and feasibility prove correct, the study is intended to inform the design of a larger multicentre clinical trial.

The Deferred Stent STEMI trial is registered at clinicaltrials.gov NCT01717573

# Rationale for a clinical event adjudication committee

As a measure of enhanced Pharmacovigilance (PV), an independent Clinical Event Committee (CEC) is proposed to review deaths (due to any cause) and specifically cardiovascular events of interest. At a high level, such events of interest will include death of any cause, non-fatal acute myocardial infarction, non-fatal stroke, hospitalization due to unstable angina, hospitalization due to heart failure and coronary revascularization procedures (i.e., percutaneous coronary intervention, coronary artery bypass grafting). The revascularization procedures will not be considered to be major adverse events of interest but will be reviewed by the CEC to ensure that events of interest (e.g., acute myocardial infarction, hospitalization for unstable angina) have not been missed.

(Note: CV events are not a defined endpoint in the Deferred Stent STEMI study, but instead will be analyzed separately within the context of standard safety analyses for the study reports and submission).

The CEC will review cases of interest to determine if they meet accepted diagnostic criteria. Causality assessments will not be made by the CEC, nor will the committee possess governance authority. The CEC will be blinded regarding any information relating to the randomization group, accepting that the trial involves an unblinded treatment assignment which may not be possible to blind for events related to the index and deferred procedures.

All deaths and pre-specified major adverse cardiovascular events (i.e., “MACE”-type events) will be prospectively collected by investigators and classified independently by the CEC. Details on these pre-specified events are listed in section 4.

As noted above, events of interest will be identified primarily by the investigator, who may use an eCRF checkbox to mark any event as a “CV event of interest”. As a conservative measure, safety data will also be reviewed by the Pharmacovigilance team in the Robertson Centre for Biostatistics, an NIHR-approved Trials Unit, in order to identify any cases which may have been missed by the investigators (for further details, please refer to Safety Monitoring in the protocol).

All organizational and operational aspects of the CEC will be administered and directed by the National Waiting Times Board (NWTB) which is the Sponsor.

# Objective of the CEC Charter

The purpose of this document is to delineate the roles, responsibilities and procedures in regards to the adjudication of cardiovascular events occurring in the Deferred Stent STEMI trial.

# Composition and responsibilities of the CEC

The CEC consists of at least 3 cardiovascular physicians who have expertise in the diagnosis and treatment of cardiovascular disorders and in the medical aspects of clinical trials:

| CEC Member | Affiliation |
| --- | --- |
| Dr John Irving (Consultant Cardiologist), Chairman | Ninewells Hospital, Dundee, Scotland |
| Dr Adelle Dawson (Consultant Cardiologist) | Ninewells Hospital, Dundee, Scotland |
| Dr Stuart Hutcheon (Consultant Cardiologist) | Ninewells Hospital, Dundee, Scotland |

In the event that a CEC member is unable to continue participation, the CEC Chairman will recommend a replacement to the Sponsor. The Sponsor has the final decision as to the replacement. CEC members may not participate in the study as principal or co-investigators, nor can they participate in the medical care of a patient in the study.

The CEC Chairman (Dr John Irving) will be responsible for:

Acting as the primary liaison between the CEC and the Sponsor

Selection of CEC members

The overall conduct of the CEC

Participating in the development of CEC Charter

Submission of General Event Forms and Death Event Forms to Sponsor and Clinical Trial Unit

CEC members will be responsible for:

Reading and understanding the relevant content of Deferred Stent STEMI pilot study (NCT 01717573)

Reviewing the relevant de-identified clinical data about a subject identified as having experienced a suspected event of interest requiring adjudication

Adjudicating pre-specified clinical events of interest (see section 4) in keeping with the study definitions outlined in section 5.

Completion of General Event Forms and Death Event Forms

Timely submission of event adjudication decisions

Communicating with the CEC Chairman about needs when necessary

Attending scheduled CEC meetings throughout the study

Completion of confidentiality form

## CEC Coordinator

The CEC is assisted by a CEC coordinator (Dr David Carrick, BHF Cardiovascular Research Centre, University of Glasgow; david.carrick@nhs.net) who is a registered physician based in the University of Glasgow and Golden Jubilee National Hospital and who has considerable previous experience in the conduct of cardiovascular clinic trial activity.

The CEC coordinator will:

Assist with preparation for the CEC meetings

Enter the classification verdicts reached at CEC meetings into the database

Interact with the CEC Chair as appropriate

# Events to be reviewed by the CEC

## 3.1 Deaths

The CEC will review all reported deaths and classify the cause of death according to the following schema:

Non-cardiovascular

A definite non-cardiovascular cause of death must be identified.

Cardiovascular (CV)

Death due to acute myocardial infarction

Death due to stroke

Sudden cardiac death

Other CV death (e.g., heart failure, pulmonary embolism, cardiovascular procedure-related)

Undetermined cause of death (i.e., cause of death unknown)

## 3.2 Non-fatal cardiovascular events

The CEC will review and adjudicate the following reported non-fatal cardiovascular events:

Acute myocardial infarction

Hospitalization for unstable angina/other angina*/chest pain*

Stroke/TIA/Other cerebrovascular events (i.e., subdural/extradural hemorrhage)**

Heart failure requiring hospitalization

Coronary revascularization procedures (i.e., percutaneous coronary intervention, coronary artery bypass grafting)***

Renal failure (>25% rise in creatinine from baseline or an absolute increase in serum creatinine of 0·.5 mg/dL (44 µmol/L) after a radiographic examination using a contrast agent (Barrett NEJM 2006;354:379-86)

Bleeding according to the ACUITY criteria (Stone Am Heart J 2004;148:764-75)

Note: Other non-fatal cardiovascular events will not routinely be reviewed by the CEC. These events will be reviewed by trained and qualified clinical research staff in the Golden Jubilee National Hospital to ensure that potential cardiovascular events requiring adjudication are not missed. If the review suggests that a potential cardiovascular event requiring adjudication may have been missed, further information will be requested, as required and, if necessary, the event will be allocated to the CEC for adjudication.

*Hospitalization for other angina or for chest pain are not study events of interest but such events will be reviewed by the CEC to ensure that acute myocardial infarction or hospitalization for unstable angina events have not been missed.

**TIAs and other cerebrovascular events (subdural hemorrhage, extradural hemorrhage) are not study events of interest but will be reviewed by the CEC to ensure that stroke events have not been missed.

***Coronary revascularization procedures (i.e., percutaneous coronary intervention, coronary artery bypass grafting) are not study events of interest but will be reviewed by the CEC to sure that study events of interest (e.g., acute myocardial infarction, hospitalization for unstable angina) have not been missed.

# Adverse Event definitions

For those event-types requiring adjudication, each event will usually be adjudicated on the basis of strict application of the endpoint definitions below. However, the clinical likelihood that a suspected event has occurred will be individually assessed even in the absence of fulfillment of all of the criteria specified in the event-definition, recognizing that information may at times be difficult to interpret (e.g., the exact measurement of ECG changes may be imprecise) or unavailable. The CEC will discuss such cases at a full CEC meeting and adjudicate them using their clinical expertise and the totality of the evidence before arriving at a classification decision that is based on full consensus.

Overall, event definitions should align with the "Standardized definitions for endpoint events in cardiovascular trials' Hicks KA et al May 2011 and the "Third Universal Definition of Myocardial Infarction" Thygesen et al Eur Heart J 2012.

## 4.1 Deaths

In cases where a patient experiences an event and later dies due to that event, the event causing death and the death will be considered as separate events *only* if they are separated by a change in calendar day. If the event causing death and the death occur on the same calendar day, death will be the only event classified.

## 4.1.1 Cardiovascular deaths

**Cardiovascular death** includes death resulting from an acute myocardial infarction, sudden cardiac death, death due to heart failure, death due to stroke and death due to other cardiovascular causes as follows:

**Death due to Acute Myocardial Infarction** refers to a death usually occurring up to 30 days after a documented acute myocardial infarction (verified either by the diagnostic criteria outlined below for acute myocardial infarction, above, or by autopsy findings showing recent myocardial infarction or recent coronary thrombus) due to the myocardial infarction or its immediate consequences (e.g., progressive heart failure) and where there is no conclusive evidence of another cause of death.

If death occurs before biochemical confirmation of myocardial necrosis can be obtained, adjudication should be based on clinical presentation and other (e.g., ECG, angiographic, autopsy) evidence.

NOTE: This category will include sudden cardiac death, involving cardiac arrest, often with symptoms suggestive of myocardial ischemia, and accompanied by presumably new ST elevation*, or new left bundle branch block*, or evidence of fresh thrombus in a coronary artery by coronary angiography and/or at autopsy, but death occurring before blood samples could be obtained, or at a time before the appearance of cardiac biomarkers in the blood (i.e., myocardial infarction Type 3 – see section 4.2.1, below).

*If ECG tracings are not available for review, the CEC may adjudicate on the basis of reported new ECG changes that have been clearly documented in the case records or in the case report form.

Death resulting from a procedure to treat an acute myocardial infarction [percutaneous coronary intervention (PCI), coronary artery bypass graft surgery (CABG)], or to treat a complication resulting from acute myocardial infarction, should also be considered death due to acute myocardial infarction.

Death resulting from a procedure to treat myocardial ischemia (angina) or death due to an acute myocardial infarction that occurs as a direct consequence of a cardiovascular investigation/procedure/operation that was not undertaken to treat an acute myocardial infarction or its complications should be considered as a death due to other cardiovascular causes.

**Sudden Cardiac Death** refers to a death that occurs unexpectedly in a previously stable patient. The cause of death should not be due to another adjudicated cause (e.g., acute myocardial infarction Type 3 – see section 4.2.1 below).

The following deaths should be included.

a. Death witnessed and instantaneous without new or worsening symptoms

b. Death witnessed within 60 minutes of the onset of new or worsening symptoms unless a cause other than cardiac is obvious.

c. Death witnessed and attributed to an identified arrhythmia (e.g.,, captured on an ECG recording, witnessed on a monitor), or unwitnessed but found on implantable cardioverter-defibrillator review.

d. Death in patients resuscitated from cardiac arrest in the absence of pre-existing circulatory failure or other causes of death, including acute myocardial infarction, and who die (without identification of a non-cardiac etiology) within 72 hours or without gaining consciousness; similar patients who died during an attempted resuscitation.

Unwitnessed death without any other cause of death identified (information regarding the patient’s clinical status in the 24 hours preceding death should be provided, if available)

**Death due to Heart Failure** refers to a death occurring in the context of clinically worsening symptoms and/or signs of heart failure without evidence of another cause of death (e.g., acute myocardial infarction).

Death due to heart failure should include sudden death occurring during an admission for worsening heart failure as well as death from progressive heart failure or cardiogenic shock following implantation of a mechanical assist device.

New or worsening signs and/or symptoms of heart failure include any of the following:

**a**. New or increasing symptoms and/or signs of heart failure requiring the initiation of, or an increase in, treatment directed at heart failure or occurring in a patient already receiving maximal therapy for heart failure

Note: If time does not allow for the initiation of, or an increase in, treatment directed at heart failure or if the circumstances were such that doing so would have been inappropriate (e.g., patient refusal), the CEC will adjudicate on clinical presentation and, if available, investigative evidence.

**b**. Heart failure symptoms or signs requiring continuous intravenous therapy (i.e., at least once daily bolus administration or continuous maintenance infusion) or chronic oxygen administration for hypoxia due to pulmonary edema.

**c**. Confinement to bed predominantly due to heart failure symptoms.

**d**. Pulmonary edema sufficient to cause tachypnea and distress **not** occurring in the context of an acute myocardial infarction, worsening renal function (that is not wholly explained by worsening heart failure/cardiac function) or as the consequence of an arrhythmia occurring in the absence of worsening heart failure.

**e**. Cardiogenic shock **not** occurring in the context of an acute myocardial infarction or as the consequence of an arrhythmia occurring in the absence of worsening heart failure.

Cardiogenic shock is defined as systolic blood pressure (SBP) < 90 mm Hg for greater than 1 hour, not responsive to fluid resuscitation and/or heart rate correction, and felt to be secondary to cardiac dysfunction and associated with at least one of the following signs of hypoperfusion:

Cool, clammy skin ***or***

Oliguria (urine output < 30 mL/hour) ***or***

Altered sensorium ***or***

Cardiac index < 2.2 L/min/m^2^

Cardiogenic shock can also be defined if SBP < 90 mm Hg and increases to ≥ 90 mm Hg in less than 1 hour with positive inotropic or vasopressor agents alone and/or with mechanical support.

**Death due to Stroke** refers to death after a documented stroke (verified by the diagnostic criteria outlined below for stroke or by typical post mortem findings) that is either a direct consequence of the stroke or a complication of the stroke and where there is no conclusive evidence of another cause of death.

NOTE: In cases of early death where confirmation of the diagnosis cannot be obtained, the CEC may adjudicate based on clinical presentation alone.

Death due to a stroke reported to occur as a direct consequence of a cardiovascular investigation/procedure/operation will be classified as death due to other cardiovascular cause.

Death due to subdural or extradural hemorrhages will be adjudicated (based on clinical signs and symptoms as well as neuroimaging and/or autopsy) and classified separately by the CV-EAC.

**Death due to Other Cardiovascular Causes** refers to a cardiovascular death not included in the above categories [e.g. pulmonary embolism, cardiovascular intervention (other than one performed to treat an acute myocardial infarction or a complication of an acute myocardial infarction – see definition of death due to myocardial infarction, above), aortic aneurysm rupture, or peripheral arterial disease]. Mortal complications of cardiac surgery or non-surgical revascularization should be classified as cardiovascular deaths.

## 4.1.2 Non-cardiovascular deaths

A non-cardiovascular death is defined as any death that is not thought to be due to a cardiovascular cause. There should be unequivocal and documented evidence of a non-cardiovascular cause of death.

Further sub-classification of non-cardiovascular death will be as follows:

Pulmonary

Renal

Gastrointestinal

Infection (includes sepsis)

Non-infectious (e.g.,, systemic inflammatory response syndrome (SIRS))

Malignancy

Hemorrhage, not intracranial

Accidental/Trauma

Suicide

Non-cardiovascular surgery

Other non-cardiovascular, specify: ________________

## 4.1.3 Undetermined cause of death

This refers to any death not attributable to one of the above categories of cardiovascular death or to a non-cardiovascular cause (e.g., due to lack of information such as a case where the only information available is “patient died”). It is expected that every effort will be made to provide the adjudicating committee with enough information to attribute deaths to either a cardiovascular or non-cardiovascular cause so that the use of this category is kept to a minimal number of patients.

## 4.1.4 Non-fatal Cardiovascular Events

Date of onset

For purposes of classification, when classifying events that are a cause of hospitalization, the date of admission will be used as the onset date. In cases where the stated date of admission differs from the date the patient first presented to hospital with the event (e.g., because of a period of observation in an emergency department, medical assessment unit or equivalent), the date of initial presentation to hospital will be used (provided that the patient had not been discharged from hospital in the interim).

For events where an admission date is not applicable (or not available), the date of onset as stated by the investigator will be used.

## 4.2.1 Acute myocardial infarction

Note on biomarker elevations:

For cardiac biomarkers, laboratories should report an upper reference limit (URL). If the 99th percentile of the upper reference limit (URL) from the respective laboratory performing the assay is not available, then the URL for myocardial necrosis from the laboratory should be used. If the 99th percentile of the URL or the URL for myocardial necrosis is not available, the MI decision limit for the particular laboratory should be used as the URL.

## Spontaneous acute myocardial infarction:

A rise and/or fall of cardiac biomarkers (troponin or CK-MB) should usually be detected (see note below) with at least one value above the upper reference limit (URL) together with evidence of myocardial ischemia with at least one of the following:

- Clinical presentation consistent with ischemia
- ECG evidence of acute myocardial ischemia (as outlined in Table 1, below) or new left bundle branch block (LBBB).
- Development of pathological Q waves on the ECG (see Table 2, below)
- Imaging evidence of new loss of viable myocardium or new regional wall motion abnormality
- Autopsy evidence of acute myocardial infarction
- If biomarkers are elevated from a prior infarction, then a spontaneous myocardial infarction is defined as:

**a**. One of the following:

o Clinical presentation consistent with ischemia

o ECG evidence of acute myocardial ischemia (as outlined in Table 1, below) or new left bundle branch block. [The events committee will adjudicate in the context of the sequential ECG changes that are commonly seen in acute ST elevation/acute non-ST elevation myocardial infarction.]

o New pathological Q waves (see Table 2, below). [The events committee will adjudicate in the context of the sequential ECG changes that are commonly seen in acute ST elevation/acute non-ST elevation myocardial infarction.]

o Imaging evidence of new loss of viable myocardium or new regional wall motion abnormality

o Autopsy evidence of acute myocardial infarction

AND

**b**. Both of the following:

o Evidence that cardiac biomarker values were decreasing (e.g., two samples 3-6 hours apart) prior to the suspected acute myocardial infarction*

o ≥ 20% increase (and > URL) in troponin or CK-MB between a measurement made at the time of the initial presentation with the suspected recurrent myocardial infarction and a further sample taken 3-6 hours later

- *If biomarkers are increasing or peak is not reached, then a definite diagnosis of recurrent myocardial infarction is generally not possible.

Percutaneous coronary intervention-related acute myocardial infarction

Peri-percutaneous coronary intervention (PCI) acute myocardial infarction is defined by any of the following criteria. Symptoms of cardiac ischemia are not required.

Biomarker elevations within 48 hours of PCI:

• Troponin or CK-MB (preferred) > 5 x URL ***and***

• No evidence that cardiac biomarkers were elevated prior to the procedure;

OR

• Both of the following must be true:

o ≥ 50% increase in the cardiac biomarker result

o Evidence that cardiac biomarker values were decreasing (e.g., two samples 3-6 hours apart) prior to the suspected acute myocardial infarction

New pathological Q waves or new left bundle branch block (LBBB).

[If the PCI was undertaken in the context of an acute myocardial infarction, the events committee will adjudicate in the context of the sequential ECG changes that are commonly seen in acute ST elevation/acute non-ST elevation myocardial infarction.]

Autopsy evidence of acute myocardial infarction

Coronary artery bypass grafting–related acute myocardial infarction

Peri-coronary artery bypass graft surgery (CABG) acute myocardial infarction is defined by the following criteria. Symptoms of cardiac ischemia are not required.

Biomarker elevations within 72 hours of CABG:

• Troponin or CK-MB (preferred) > 10 x URL ***and***

• No evidence that cardiac biomarkers were elevated prior to the procedure;

OR

• Both of the following must be true:

o ≥ 50% increase in the cardiac biomarker result

o Evidence that cardiac biomarker values were decreasing (e.g., two samples 3-6 hours apart) prior to the suspected acute myocardial infarction

AND

One of the following:

New pathological Q-waves (preferably with evidence of persistence)

[If the CABG was undertaken in the context of an acute myocardial infarction, the events committee will adjudicate in the context of the sequential ECG changes that are commonly seen in acute ST elevation/acute non-ST elevation myocardial infarction.]

New LBBB (preferably with evidence of persistence)

Angiographically documented new graft or native coronary artery occlusion

Imaging evidence of new loss of viable myocardium

OR

Autopsy evidence of acute myocardial infarction

**Note:** For a diagnosis of acute myocardial infarction, a rise and/or fall of cardiac biomarkers should usually be detected. However, myocardial infarction may be adjudicated for an event that has characteristics which are very suggestive of acute infarction but which does not meet the strict definition because biomarkers are not available (e.g., not measured) or are non-contributory (e.g., may have normalized).

Suggestive characteristics are:

Typical cardiac ischemic-type pain/discomfort
(except for suspected acute myocardial infarction occurring in the context of PCI or CABG where this requirement need not apply)

AND

New ECG changes* or other evidence to support a diagnosis of acute myocardial infarction (e.g., imaging evidence of new loss of viable myocardium/new regional wall motion abnormality or angiography demonstrating occlusive coronary thrombus)

*If ECG tracings are not available for review, the CEC may adjudicate on the basis of reported ECG changes that have been clearly documented in the case records or in the case report form.

Clinical classification of different types of myocardial infarction

Myocardial infarctions will be clinically classified as:

Type 1

Spontaneous myocardial infarction related to ischemia due to a primary coronary event such as plaque erosion and/or rupture, fissuring, or dissection.

Type 2

Myocardial infarction secondary to ischemia due to either increased oxygen demand or decreased supply, e.g. coronary artery spasm, coronary embolism, anemia, arrhythmias, hypertension, or hypotension.

Type 3

Sudden unexpected cardiac death, including cardiac arrest, often with symptoms suggestive of myocardial ischemia, accompanied by presumably new ST elevation, or new LBBB, or evidence of fresh thrombus in a coronary artery by angiography and/or at autopsy, but death occurring before blood samples could be obtained, or at a time before the appearance of cardiac biomarkers in the blood.

Type 4a

Myocardial infarction associated with PCI.

Type 4b

Myocardial infarction associated with stent thrombosis as documented by angiography or at autopsy.

Type 5

Myocardial infarction associated with CABG.

Myocardial infarctions will be further sub-classified as:

ST segment elevation myocardial infarction (STEMI).
**or**

Non-ST segment elevation myocardial infarction (NSTEMI).
**or**

Myocardial infarction, type (i.e., STEMI or NSTEMI) unknown.

| Table 1: ECG manifestations of acute myocardial ischemia (in absence of left ventricular hypertrophy and left bundle branch block) |
| --- |
| ST elevation  New ST elevation at the J-point in two anatomically contiguous leads with the cut-off  points: ≥ 0·2 mV in men (> 0·25 mV in men < 40 years) or ≥ 0·15 mV in women in leads V2-V3 and/or ≥ 0·1 mV in other leads.  ST depression and T wave changes  New horizontal or down-sloping ST depression ≥ 0·05 mV in two  contiguous leads; and/or new T wave inversion ≥ 0·1 mV in two contiguous  leads.  The above ECG criteria illustrate patterns consistent with myocardial ischemia. In patients with abnormal biomarkers, it is recognized that lesser ECG abnormalities may represent an ischemic response and may be accepted under the category of abnormal ECG findings. |

| Table 2: Pathological Q waves: |
| --- |
| Any Q-wave in leads V2-V3 ≥ 0·02 seconds or QS complex in leads V2 and V3  Q-wave ≥ 0·03 seconds and ≥ 0·1 mV deep or QS complex in leads I, II, aVL, aVF, or V4-V6 in any two leads of a contiguous lead grouping (I, aVL, V6; V4-V6; II, III, and aVF) a  A The same criteria are used for supplemental leads V7-V9, and for the Cabrera frontal plane lead grouping. |

## 4.2.2 Hospitalization for unstable angina

For the diagnosis of hospitalization due to unstable angina there should be emergency/unplanned admission to a hospital setting (emergency room, observation or inpatient unit) that results in at least one overnight stay (i.e., a date change) with fulfillment of the following criteria:

There should be:

1. Cardiac ischemic-type symptoms at rest (chest pain or equivalent) or an accelerating pattern of angina (e.g., exercise-related ischemic-type symptoms increasing in frequency and/or severity, decreasing threshold for onset of exercise related ischemic type symptoms) but without the fulfillment of the above diagnostic criteria for acute myocardial infarction.

and

2 The need for treatment with parenteral (intravenous, intra-arterial, buccal, transcutaneous, or subcutaneous) anti-ischemic/antithrombotic therapy and/or coronary revascularization.

and

3a ECG manifestations of acute myocardial ischemia (New ST-T changes meeting the criteria for acute myocardial ischemia - as outlined in Table 1, section 5.2.1).

or

3b Angiographically significant coronary artery disease thought to be responsible for the patient’s presentation. [If both invasive and CT angiographic imaging of the coronary arteries were performed, the results of the invasive coronary angiogram should take preference.]

and

4 The CEC should be satisfied that unstable angina was the primary reason for hospitalization.

## 4.2.3 Hospitalization for other angina*

For the diagnosis of hospitalization for other angina, there should be emergency/unplanned admission to a hospital setting (emergency room, observation or inpatient unit) that results in at least one overnight stay (i.e., a date change) with fulfillment of the following criteria:

There should be:

Typical cardiac ischemic-type symptoms but without the fulfillment of the above diagnostic criteria for acute myocardial infarction or unstable angina.

and

2 The need for treatment with new or increased antianginal therapy (excluding sublingual nitrate therapy).

and

3a Investigations undertaken in view of the event (e.g., exercise ECG or stress myocardial perfusion scan) showing evidence of reversible myocardial ischemia.

or

3b Coronary angiography showing angiographically significant coronary disease thought to be responsible for the patient’s presentation. [If both invasive and CT angiographic imaging of the coronary arteries were performed, the results of the invasive coronary angiogram should take preference.]

and

4 The CEC should be satisfied that angina was the primary reason for hospitalization.

## 4.2.4 Hospitalization for other chest pain*

There should be:

Emergency/unplanned admission to a hospital setting (emergency room, observation or inpatient unit) that results in at least one overnight stay i.e. a date change) due to chest pain but where the definitions (above) of acute myocardial infarction, hospitalization for unstable angina or hospitalization for other angina are not met.

The CEC should be satisfied that chest pain was the primary reason for hospitalization.

*These events are not study cardiovascular events of interest but the definitions provided for these events will be used by the CEC to categorize reported myocardial infarction, angina and chest pain events that do not meet the study definition of acute myocardial infarction or hospitalization for unstable angina.

## 4.2.5 Stroke

**Stroke** is defined as an acute episode of neurological dysfunction caused by focal or global brain, spinal cord, or retinal vascular injury.

**A** For the diagnosis of stroke, the following 4 criteria should usually be fulfilled:

1. Rapid onset* of a focal/global neurological deficit with at least one of the following:

Change in level of consciousness

Hemiplegia

Hemiparesis

Numbness or sensory loss affecting one side of the body

Dysphasia/aphasia

Hemianopia (loss of half of the field of vision of one or both eyes)

Complete/partial loss of vision of one eye

Other new neurological sign(s)/symptom(s) consistent with stroke

*If the mode of onset is uncertain, a diagnosis of stroke may be made provided that there is no plausible non-stroke cause for the clinical presentation.

2. Duration of a focal/global neurological deficit > 24 hours

or

< 24 hours if

(i) this is because of at least one of the following therapeutic interventions:

(a) pharmacologic i.e. thrombolytic drug administration.

(b) non-pharmacologic i.e. neurointerventional procedure (e.g., intracranial angioplasty).

or

(ii) brain imaging available clearly documenting a new hemorrhage or infarct.

or

(iii) the neurological deficit results in death

3. No other readily identifiable non-stroke cause for the clinical presentation (e.g., brain tumor, hypoglycemia, peripheral lesion).

4. Confirmation of the diagnosis by at least one of the following**:

neurology or neurosurgical specialist.

brain imaging procedure (at least one of the following):

CT scan.

MRI scan.

cerebral vessel angiography.

lumbar puncture (i.e., spinal fluid analysis diagnostic of intracranial hemorrhage).

******If a stroke is reported but evidence of confirmation of the diagnosis by the methods outlined above is absent, the event will be discussed at a full CEC meeting. In such cases, the event may be adjudicated as a stroke on the basis of the clinical presentation alone but *full CEC consensus will be mandatory.*

B If the acute neurological deficit represents a worsening of a previous deficit, this worsened deficit must have:

Persisted for more than one week

**Or** < one week if

(i) this is because of at least one of the following therapeutic interventions:

(a) pharmacologic i.e. thrombolytic drug administration.

(b) non-pharmacologic i.e. neurointerventional procedure (e.g., intracranial angioplasty).

or

(ii) brain imaging available clearly documenting an appropriate new CT/MRI finding.

or

(iii) the neurological deficit results in death

Strokes will be further sub-classified as:

Ischemic (non-hemorrhagic) stroke

(i.e., caused by an infarction of central nervous system tissue)

or

Hemorrhagic stroke***

(i.e., caused by nontraumatic intraparenchymal, intraventricular, or subarachnoid hemorrhage)

or

Stroke type (i.e., hemorrhagic or ischemic) unknown (i.e., when imaging/other investigations are unavailable or inconclusive).

***Subdural and extradural hemorrhages will be adjudicated (based on clinical signs and symptoms as well as neuroimaging and/or autopsy) and classified separately by the CEC

## 4.2.6. Heart Failure requiring hospitalization

For the diagnosis of heart failure requiring hospitalization, there should be emergency/unplanned admission to a hospital setting (emergency room, observation or inpatient unit) that results in at least one overnight stay (i.e., a date change) with fulfillment of the following criteria:

There should be:

Clinical manifestations of new or worsening heart failure including at least one of the following:

New or worsening dyspnea on exertion

New or worsening dyspnea at rest

New or worsening fatigue/decreased exercise tolerance

New or worsening orthopnea

New or worsening PND (paroxysmal nocturnal dyspnea)

New or worsening lower limb or sacral edema

New or worsening pulmonary crackles/crepitations

New or worsening elevation of JVP (jugular venous pressure)

New or worsening third heart sound or gallop rhythm

**And**

1. Investigative evidence of structural or functional heart disease (if available) with at least *one* of the following:

Radiological evidence of pulmonary edema/congestion or cardiomegaly.

Imaging ( e.g. echocardiography, cardiac magnetic resonance imaging, radionuclide ventriculography) evidence of an abnormality (e.g., left ventricular systolic dysfunction, significant valvular heart disease, left ventricular hypertrophy).

- - Elevation of BNP or NT-proBNP levels.
  - Other investigative evidence of structural or functional heart disease (e.g., evidence obtained from pulmonary artery catheterization).

**And**

**3** Need for new/increased therapy***** specifically for the treatment of heart failure

including at least one of the following:

New or increased oral therapy for the treatment of heart failure

(See note on oral therapy, below)

Initiation of intravenous diuretic, inotrope, vasodilator or other recognized intravenous heart failure treatment or up-titration of such intravenous therapy if already receiving it

Mechanical or surgical intervention (e.g., mechanical or non-invasive ventilation, mechanical circulatory support, heart transplantation, ventricular pacing to improve cardiac function), or the use of ultrafiltration, hemofiltration, dialysis or other mechanical or surgical intervention that is specifically directed at treatment of heart failure.

Note on oral therapy: In general, for an event to qualify as *heart failure requiring hospitalization* on the basis of *oral* heart failure therapy (i.e., in cases where none of the non-pharmacological treatment modalities listed above have been utilized), the new or increased oral therapy should include oral diuretics. However, in special cases, other new or increased oral therapy (e.g.,, hydralazine/long-acting nitrate, aldosterone antagonist) may be accepted provided that the adjudication committee is satisfied that:

the new or increased oral therapy was primarily directed at treating clinical manifestations of new or worsening heart failure (rather than, for example, initiation or up-titration of heart failure therapy as part of the routine optimization of medical therapy)

and

the totality of the evidence indicates that heart failure, rather than any other disease process, was the primary cause of the clinical presentation.

*****If time does not allow for the initiation of, or an increase in, treatment directed at heart failure or if the circumstances were such that doing so would have been inappropriate (e.g., patient refusal), the CEC will adjudicate on clinical presentation and, if available, investigative evidence.

and

**4** The CEC should be satisfied that heart failure was the primary disease process accounting for the clinical presentation.

## 4.2.7. Renal Failure requiring hospitalization

Contrast-induced nephropathy: is defined as either a greater than 25% increase of serum creatinine or an absolute increase in serum creatinine of 0·5 mg/dL after a radiographic examination using a contrast agent.

## 4.2.8. Bleeding requiring hospitalization

Bleeding: is defined according to the ACUITY criteria: major bleed = intracranial or intraocular bleeding; bleeding at the site of angiography requiring intervention; a hematoma of 5 cm in diameter; a reduction in hemoglobin level of at least 4 g/dL in the absence of overt bleeding or 3 g/dL with a source of bleeding; or transfusion.

# Adjudication process

A flowchart of the overall CEC adjudication process is shown in Appendix A.

For the first 10 reported events requiring adjudication, the events will be reviewed at a CEC meeting with at least 3 members present. The purpose of this committee review will be to ensure that all committee members are applying the endpoint definitions as described in this charter and that all members are aligned in their applications of the definitions to the classifications of events. In this review of the initial 10 events, full consensus will be required for each final classification decision, as noted in section 6.4 below.

## 6.1 Event identification

The Deferred Stent STEMI study will use paper-based and electronic data capture (EDC). Those events requiring review by the CEC (see section 4) will be reported by the Investigator via the EDC (electronic data capture) system. The Investigator will complete all required Case Report Form pages for the event type.

As a conservative measure, safety data will also be reviewed on behalf of the sponsor by the Pharmacovigilance Office of the NIHR Clinical Trials Unit (Robertson Centre for Biostatistics, University of Glasgow).

## 6.2 Phase 1 CEC review

The CEC members will receive electronic notification that they have events ready for adjudication. The date of dispatch to the CEC members will be recorded electronically.

With the exception of possible cerebrovascular events (see note below), each suspected event package will be reviewed independently by 2 of the CEC members with an interest in cardiology (i.e., a pair selected from the CEC). Pairs will be rotated automatically in a manner that ensures that events are distributed to the members on an even basis. The pair will enter their adjudication decisions into the General Event Classification Form. For each event where the reviewers have agreed on a classification, the event is deemed classified. Disagreements will be highlighted for adjudication at a scheduled CEC meeting (“Phase 2 CEC review” - see section 6.4). The decision to defer classification until a scheduled CEC meeting will be logged.

**Note:** All possible cerebrovascular events will be classified by consensus at a CEC meeting with all members (ideally including a physician with experience in cerebrovascular medicine) present.

## 6.3 Incomplete event data

If, having reviewed the event data pertaining to an event, a CEC member deems that the information provided is insufficient for the purposes of event adjudication, an electronic request for further information detailing the information required will be made. The date of request will be recorded electronically and the event will be classified as not adjudicated/pending additional information. When new information becomes available, it will be sent in a deidentified form to the CEC Chair. It is expected that both the details of the original request for information and the new or updated information received will be clearly flagged to the adjudicators within the event package.

In instances where it is confirmed that efforts to obtain requested information have been unsuccessful (e.g., because the study site has indicated that the information is not available), classification of the event will be deferred pending its discussion at a scheduled CEC meeting (see section 6.4).

## 6.3 Phase 2 CEC review

The CEC will convene at regular intervals throughout the study. In general, these will be face- to- face meetings, however, if for some reason a face- to- face meeting is not possible, a meeting by teleconference may substitute.

The frequency of meetings depends on the quantity of clinical events received by the CEC but it is planned that they will be scheduled to occur once quarterly. These may be cancelled if there is no business for discussion or cases to be reviewed by full committee.

The primary objective of CEC meetings is the “Phase 2 review” and classification of those events for which a final classification decision has not been achieved by the Phase 1 review process already outlined above and to review and classify cerebrovascular events that have been reported. Phase 2 review of an event constitutes the discussion and adjudication of the event by the CEC as a group.

For cerebrovascular events, as well as all other events, the final classification decision will be decided on the basis of full CEC consensus.

If the CEC are unable to arrive at a classification verdict for an event because of incomplete or inadequate information and it is felt that such information may be obtainable (i.e., the study site has *not* indicated that the information required is unavailable), the Chairman will detail the precise information/documentation that is needed to achieve classification and this will be requested using the process described above. The event will be tabled and reviewed subsequently at a CEC meeting when the information requested has been made available (or, when, despite best efforts, it is confirmed that the information will not be obtainable).

## 6.4 Adjudication timelines

The CEC will make every effort to review events and to enter their classification decisions onto the General Event Classification Forms within 2 to 4 weeks from the time that the event data is received by the CEC members, although this may vary slightly. To facilitate the prompt adjudication of events, it is expected that event data received by the CEC will be as clean and complete as possible and that any CEC data-queries are resolved in a timely fashion.

Every effort will be made to ensure that scheduled CEC meetings take place at least quarterly. The frequency of CEC meetings may be increased if required; provided that there is mutual agreement between the Sponsor and the CEC before any change is made.

If necessary, the above timelines may be amended as the study progresses, if the CEC and the other relevant parties agree on a new schedule of event turn-around time.

## 6.5 Interactions with Sponsor and Communications

The CEC will make every reasonable effort to answer Sponsor queries and provide medical advice if requested, as well as any reasonable request for a periodic review meeting.

The primary point of contact between the CEC and Sponsor will be the trial PI (Professor Colin Berry).

# Clinical data to be provided

The trial management team (including Prof Berry, Dr Carrick, Ms Joanne Kelly CRN) will provide event data for each potential cardiovascular event requiring adjudication to the CEC. These events will also be provided to the Pharmacovigilance Office of the NIHR Trials Unit. The CEC will be blinded to all patient randomization schedules. For studies that are open label, such as the Deferred Stent STEMI study, treatment allocations will be blinded by prior to documents being submitted for CEC review and classifications.

Data to be included for event classification will include:

Subject study identification number and event details

Adverse event form

On request: Relevant de-identified CRF data (including any relevant event-specific CRFs e.g. the *myocardial infarction/hospitalization for unstable angina/other angina/*

*chest pain* event form).

Supportive source documentation as required

Baseline and subsequent scheduled ECGs obtained during study participation.

All clinical data would be de-identified.

De-identified Source Documentation

The following source documents (if available) will be provided to the CEC as part of the standard dossier contents for cardiovascular events requiring review/adjudication:

Death

Hospital Discharge Summary/Death Summary

Autopsy Report

Death Certificate

Admission History & Physical (if applicable)

Acute Myocardial Infarction/Hospitalization for Unstable Angina/Other Angina/Chest Pain

Hospital Discharge Summary

ECGs

Pre-Randomization/Screening

Baseline (prior to event but post-randomization)

During Event

Post-Event

Relevant Procedure/Operation Reports

Relevant Laboratory Reports (e.g., that document the cardiac enzyme/marker measurements provided – peak values and pre-procedure and post-procedure values, where applicable)

Reports for other investigations taken:

PCI Report

CABG Report

Coronary Angiography Report

Echocardiogram Report

Exercise ECG Report

Stress Myocardial Perfusion Scan Report

Other investigation report undertaken to test for presence of reversible myocardial ischemia

Admission History & Physical

Stroke/TIA/Other cerebrovascular events

Hospital Discharge Summary

Neurology Consultation Report(s)

Reports for other investigations undertaken:

CT Brain Scan Report

MRI Brain Scan Report

Cerebral Angiography Report

Lumbar Puncture Report

Admission History & Physical

Heart Failure requiring hospitalization

Hospital Discharge Summary

Chest X-Ray Report

Prescription Sheets/Medication Administration Records

Echocardiogram Report

Relevant Laboratory Reports (e.g., for peak BNP/NT-proBNP)

Reports for other investigations undertaken:

Cardiac Magnetic Resonance Imaging

Radionuclide Ventriculogram Scan

Pulmonary Artery Catheterization

Admission History & Physical

Coronary revascularization procedure

Hospital Discharge Summary

Relevant Procedure/Operation Reports

Bleeding

Hospital Discharge Summary

Relevant Procedure/Operation Reports

Hb

Blood transfusion results

Diagnostic and therapeutic procedures (e.g., gastroscopy)

# CEC Quality assurance

For the purposes of quality assurance, 10 % of all events initially classified may be subject to review by the CEC again. If there are any discrepancies between the initial and the subsequent adjudication decisions, the Chairman and the Sponsor will discuss the steps necessary to ensure reconciliation and resolution of the issue.

# Appendix A: Adjudication Process Flowcharts

## Cardiovascular Event Flowchart:

## Cerebrovascular Event Flow Chart:

# References

1. The TIMI Study Group. The Thrombolysis In Myocardial Infarction (TIMI) trial. *N Engl J Med*. 1985; **31**:932–36.

2. Gibson CM, Cannon CP, Murphy SA, et al. Relationship of TIMI myocardial perfusion grade to mortality after administration of thrombolytic drugs. *Circulation* 2000; **101**:125−30.

3. Gibson CM, Cannon CP, Daley WL, et al. TIMI frame count: a quantitative method of assessing coronary artery flow. *Circulation* 1996 ; **93**:879−88.

4. McEntegart MB, Kirtane AJ, Cristea E, et al. Intraprocedural thrombotic events during percutaneous coronary intervention in patients with non-ST-segment elevation acute coronary syndromes are associated with adverse outcomes: analysis from the ACUITY (Acute Catheterization and Urgent Intervention Triage Strategy) trial. *J Am Coll Cardiol* 2012; **59**: 1745−51.

5. Aldrich HR, Wagner NB, Boswick J, Corsa AT, Jones MG, Grande P, Lee KL, Wagner GS. Use of initial ST-segment deviation for prediction of final electrocardiographic size of acute myocardial infarcts. *Am J Cardiol* 1988; **61**:749−53.

6. Steg PG, James SK, Atar D, et al. ESC Guidelines for the management of acute myocardial infarction in patients presenting with ST-segment elevation: The Task Force on the management of ST-segment elevation acute myocardial infarction of the European Society of Cardiology (ESC). Eur Heart J 2012; 33:2569−619.

7. Wijns W, Kolh P, Danchin N, et al. Guidelines on myocardial revascularization. *Eur Heart J* 2010; **31**: 2501−55.

8. Kramer CM, Barkhausen J, Flamm SD, Kim RJ, Nagel E; Society for Cardiovascular Magnetic Resonance Board of Trustees Task Force on Standardized Protocols. Standardized cardiovascular magnetic resonance imaging (CMR) protocols, society for cardiovascular magnetic resonance: board of trustees task force on standardized protocols. *J Cardiovasc Magn Reson* 2008; **10**:35.

9. Kellman P, Arai E, McVeigh ER, Aletras AH. Phase-sensitive inversion recovery for detecting myocardial infarction using gadolinium-delayed hyperenhancement. *Magn Reson Med* 2002; **47**:372−83.

10. Berry C, Kellman P, Mancini C, Chen MY, Bandettini WP, Lowrey T, Hsu LY, Aletras AH, Arai E. Magnetic resonance imaging delineates the ischemic area at risk and myocardial salvage in patients with acute myocardial infarction. *Circ Cardiovasc Imaging* 2010; **3**:527−35.

11. Guidelines for Good Clinical Practice in Clinical Trials: <http://www.mrc.ac.uk/Utilities/Documentrecord/index.htm?d=MRC002416>.

12. Thygesen K, Alpert JS, Jaffe AS, et al. Third universal definition of myocardial infarction. *Circulation* 2012; **126**:2020−35.

13. Barrett BJ, Parfrey PS. Clinical practice. Preventing nephropathy induced by contrast medium. *N Engl J Med* 2006; **354**:379−86.

14. Stone GW, Bertrand M, Colombo A, et al. Acute Catheterization and Urgent Intervention Triage strategY (ACUITY) trial: study design and rationale. *Am Heart J* 2004;**148**:764-75.
